# Transition metal sulfides grown on graphene fibers for wearable asymmetric supercapacitors with high volumetric capacitance and high energy density

**DOI:** 10.1038/srep26890

**Published:** 2016-06-01

**Authors:** Weihua Cai, Ting Lai, Jianwei Lai, Haoting Xie, Liuzhang Ouyang, Jianshan Ye, Chengzhong Yu

**Affiliations:** 1 College of Chemistry and Chemical Engineering, South China University of Technology, Guangzhou 510641, People’s Republic of China; 2 College of Materials Science and Engineering, South China University of Technology, Guangzhou, 510641, People’s Republic of China; 3 Australian Institute for Bioengineering and Nanotechnology, The University of Queensland, Brisbane, Australia

## Abstract

Fiber shaped supercapacitors are promising candidates for wearable electronics because they are flexible and light-weight. However, a critical challenge of the widespread application of these energy storage devices is their low cell voltages and low energy densities, resulting in limited run-time of the electronics. Here, we demonstrate a 1.5 V high cell voltage and high volumetric energy density asymmetric fiber supercapacitor in aqueous electrolyte. The lightweight (0.24 g cm^−3^), highly conductive (39 S cm^−1^), and mechanically robust (221 MPa) graphene fibers were firstly fabricated and then coated by NiCo_2_S_4_ nanoparticles (GF/NiCo_2_S_4_) via the solvothermal deposition method. The GF/NiCo_2_S_4_ display high volumetric capacitance up to 388 F cm^−3^ at 2 mV s^−1^ in a three-electrode cell and 300 F cm^−3^ at 175.7 mA cm^−3^ (568 mF cm^−2^ at 0.5 mA cm^−2^) in a two-electrode cell. The electrochemical characterizations show 1000% higher capacitance of the GF/NiCo_2_S_4_ as compared to that of neat graphene fibers. The fabricated device achieves high energy density up to 12.3 mWh cm^−3^ with a maximum power density of 1600 mW cm^−3^, outperforming the thin-film lithium battery. Therefore, these supercapacitors are promising for the next generation flexible and wearable electronic devices.

Wearable technology has been the focus of significant attention for its promising application potentials in daily life^1^,^2^,^3^. However, several challenges must be overcome before wearable energy storage devices (WESD) can be commercially produced for electronic markets. One challenge for developing WESDs is the low energy density, which limits the run-time of electronics^4^,^5^,^6^,^7^. For example, electrochemical capacitors, also called supercapacitors (SCs)^8^, can provide higher power densities, faster charge/discharge rates and longer lifetimes but offer quite low energy densities^9^. Comparing the many different SC structures, fiber supercapacitors (FSCs) are of particular interest because they have smaller volumes and lighter weights, especially compared to sandwich-structure supercapacitors^10^, leading to great potential for its use in micro-devices and macro-devices including wearable smart jewelries, energy textiles, artificial skin, and stretchable sensors^11^.

Recently, considerable efforts have been made to fabricate high-performance FSCs based on graphene or carbon nanotubes (CNT). Yu *et al.*^12^ reported the fabrication of Nitrogen doped CNT-graphene fibers using silica capillary columns as reactors at 220 °C. Though the maximum volumetric capacitance of these prepared fibers can achieve up to about 300 F cm^−3^, the mechanical strength was too low (84 MPa) to be wearable. Early examples such as SWCNT@C^13^, porous graphene fibers^14^ and MoS_2_-RGO/MWCNT^15^ were also not wearable. To enhance the mechanical strength of the fibers, Liu *et al.*^16^ tried to use graphene-Ni textile as the composite electrodes and Huang *et al.*^17^ used the stainless steel yarns @ MnO_2_ @ polypyrrole (PPy) as electrodes to make wearable FSCs. However, these reported wearable FSCs, based on a symmetric device configuration with two fiber electrodes, had operating voltages limited to 0.8 V. According to the equation E_x_ = (1/2) × C_x_·Δ*E*^2^ (where E_X_ represents the energy density, C_x_ is the specific capacitance, Δ*E* is the cell voltage)^18^, the cell voltage of FSCs could be enlarged to improve energy density. In a typical asymmetric configuration, a device with metal oxide like ruthenium oxide (RuO_2_)^19^ or manganese oxide (MnO_2_)^20^ as the positive electrode and with carbon-based material as the negative electrode exhibits higher cell voltage than that of a symmetric cell. Unfortunately, as of today, there have been few reports on high cell voltage wearable asymmetric fiber supercapacitors (AFSCs).

Transition metal sulfides have been investigated as a new type electrode material for pseudocapacitors with good performance^21^,^22^. When compared to nickel sulfide and cobalt sulfide, NiCo_2_S_4_ can offer richer redox reactions than the corresponding single component sulfide^23^. In this regard, NiCo_2_S_4_ nanoparticles were considered to be excellent materials for the asymmetric supercapacitors^24^,^25^,^26^. Here, we report, for the first time, the fabrication of AFSCs with high cell voltage up to 1.5 V based on the graphene fibers (GFs) coated by nickel cobalt sulfide (NiCo_2_S_4_) nanoparticles. In the materials design, GFs synthesized by low-temperature (80 °C) reduction of graphene oxide (GO) were used as lightweight (0.24 g cm^−3^), highly conductive (39 S cm^−1^), and mechanically robust (221 MPa) electrodes. GFs were chosen as the substrate electrodes because fibers could be produced by mixing GO and ascorbic acid, making the fabrication process easy and scalable. GFs coated by NiCo_2_S_4_ nanoparticles (GF/NiCo_2_S_4_) were synthesized by a solvothermal deposition method. Importantly, the composite electrode not only maintained the mechanical strength but also allowed the potentials to construct asymmetric SCs. Therefore, the high-voltage wearable AFSCs made of GFs as the negative electrode and GF/NiCo_2_S_4_ as the positive electrode exhibited impressive performance. The resultant fiber GF/NiCo_2_S_4_ displayed a remarkable volumetric capacitance up to 388 F cm^−3^ at 2 mV s^−1^ in a three-electrode cell and 300 F cm^−3^ at 175.7 mA cm^−3^ in a two-electrode cell. The wearable AFSCs showed a high volumetric energy density up to 12.3 mWh cm^−3^, and a maximum power density of 1.6 W cm^−3^ (normalized to the active materials including two fibers), the highest value among the previous reports to date. When it was normalized to the cell stack including the two fibers, separator, and electrolyte, the stack energy density still achieved 3.0 mWh cm^−3^, which was comparable to that of the thin-film lithium battery. The AFSCs tested under different bending angles from 0 to 180° or even under water showed excellent electrochemical performance. To demonstrate the potential use in wearable applications, an AFSC ring was weaved into a textile and three AFSCs connected in series were used to power a light-emitting diode (LED). To the best of our knowledge, the volumetric energy density of the AFSCs based on GF/NiCo_2_S_4_ is among the highest performance reported for fiber-shaped wearable supercapacitors.

## Results

### Solution processed preparation of GFs

**Figure 1:**
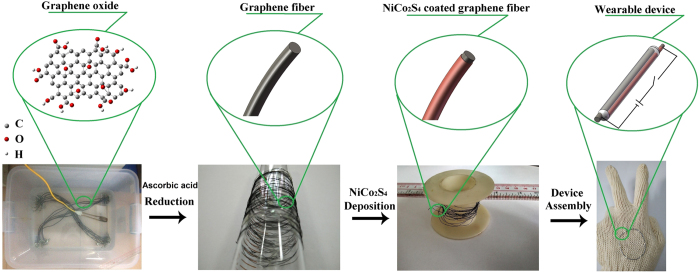
Schematic illustration of the fabrication process of wearable asymmetric fiber supercapacitors.

As shown in Fig. 1, GFs were firstly fabricated from the mixture of GO and ascorbic acid (AA) inside silicone tubing at low temperature of 80 °C in a water bath (Fig. S1). AA was used as a reducing agent in the previous report^27^ but also a gelating agent in our synthesis. After the reduction procedure, the wet GFs inside the tube were dried into solid state GFs in atmosphere.

Actually it is a scalable method to prepare GFs with the length tuned by the length of silicone tubing. The produced quantity of GFs depends both on the amount of raw materials (the mixture of GO and AA) and the length of silicone tubing. Furthermore, the diameter of GFs can be modified by varying the concentration of GO. GF-801 and GF-802 with different diameters of about 118 and 127 um, respectively (Fig. S2) were synthesized for comparisons. The as prepared GFs were lightweight and mechanically robust. For example, the density of GF-801 was only 0.24 g cm^−3^, which was only 2.7% of that of pure Ni yarns (8.91 g cm^−3^) and 1.1% of that of Pt yarns (21.45 g cm^−3^). Stress-strain tests (Table 1) showed that the GF-801 (221 MPa) were 550% stronger than pristine cotton yarns (40 MPa)^16^. The GF (>30 cm in length) was strong enough to hang a Chinese coin (Fig. S3) and the GF/NiCo_2_S_4_ (5 cm) was able to hang a balance weight (100 g) (Fig. S4). Importantly, the GFs both possessed high conductivity and maintained the textile-like flexibility. The conductivity of GF-801 and GF-802 were 39 and 26 S cm^−1^ respectively, which were similar to that of wet-spinning porous GFs. Therefore, it is easy to see that GFs would be an excellent candidate as the substrate electrodes for wearable devices.

### GF/NiCo_2_S_4_ composite electrodes

**Figure 2:**
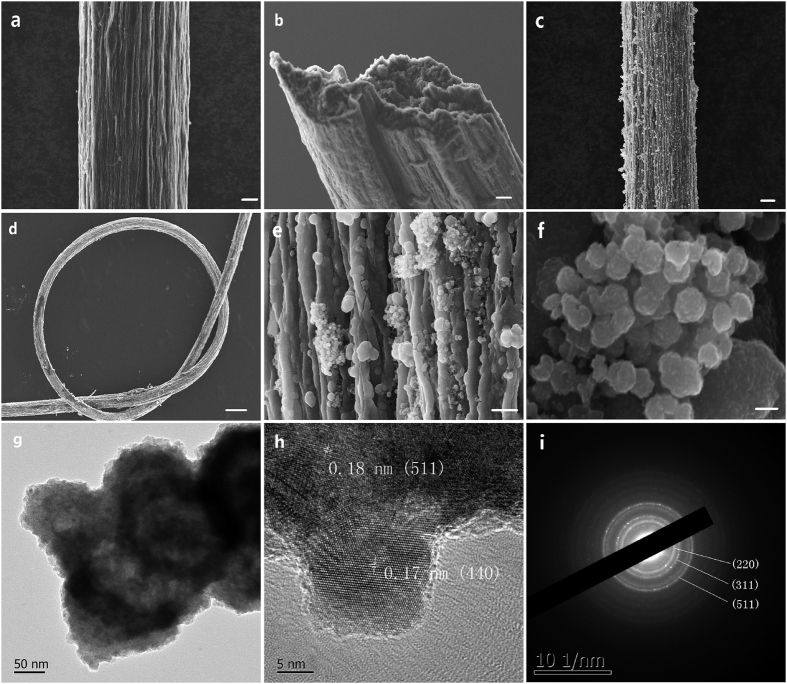
(**a**,**b**) SEM images of GF. (**c**–**f**) SEM images of GF/NiCo_2_S_4_. (**g**,**h**) TEM images of NiCo_2_S_4_. (i) SAED of NiCo_2_S_4_. Scar bar: (**a**) 10 μm; (**b**) 10 μm; (**c**) 10 μm; (**d**) 200 μm and (**e**) 1 μm.

To synthesize the composite electrode, NiCo_2_S_4_ nanoparticles were deposited onto the GFs. More specifically, GFs were coated by NiCo_2_S_4_ via a solvothermal deposition reaction in a Teflon autoclave at 180 °C for 24 h. Figure 2a–f compared the morphology structure between GFs and GF/NiCo_2_S_4_ by SEM images. Densely stacked sheets aligned along their long axis could be found in GFs (Fig. 2a) and the cross-section surface of GFs was quite porous. After the solvothermal reaction, NiCo_2_S_4_ nanoparticles were well anchored on the surface of GFs (Fig. 2c–e). The GF/ NiCo_2_S_4_ still maintained so excellent flexibility that GF/NiCo_2_S_4_ could be tied into a beautiful micro-knot (Fig. 2d). The size of the NiCo_2_S_4_ nanoparticles (Fig. 2f) varied from 150 to 200 nm. TEM image also indicated the nano-sphere morphology of NiCo_2_S_4_ nanoparticles (Fig. 2g). The high-resolution TEM image further revealed that the lattice spacing were about 0.17 and 0.18 nm, which could be assigned to (400) and (511) planes of the NiCo_2_S_4_ phase (Fig. 2h). Also, the selective-area electron diffraction (SAED) pattern (Fig. 2i) for a single nanosphere showed the ring patterns, which were well indexed to (220), (311) and (511) diffractions^28^.

**Figure 3:**
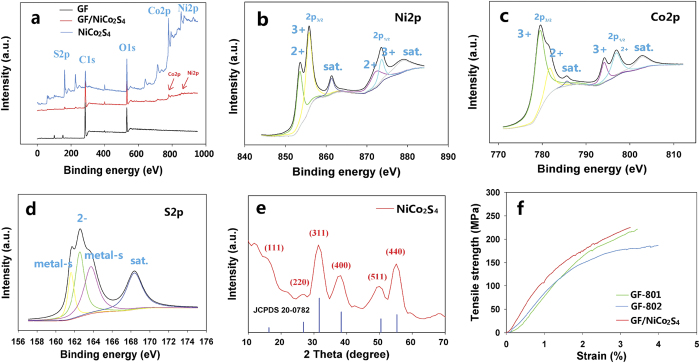
(**a**) The wide survey XPS spectrum of GF, GF/NiCo_2_S_4_ and NiCo_2_S_4_. (**b**) High-resolution Ni2p XPS spectrum of the GF/NiCo_2_S_4_. (**c**) High-resolution Co2p XPS spectrum of the GF/NiCo_2_S_4_. (**d**) High-resolution S2p XPS spectrum of the GF/NiCo_2_S_4_. (**e**) XRD spectrum of NiCo_2_S_4_. (**f**) Tensile strength curves of GF-801, GF-802, GF/NiCo_2_S_4_.

XPS survey was conducted to investigate the composition differences between GF, NiCo_2_S_4_ and GF/NiCo_2_S_4_ (Fig. 3a). The peaks at 162.4, 285.1, 532.8, 779.4, and 855.5 eV corresponding to S 2p, C 2p, O 1s, Co 2p, and Ni 2p, respectively, indicated the existence of S, C, O, Co, and Ni elements in the GF/NiCo_2_S_4_ sample. The Co 2p and Ni 2p spectra were fitted by using a Gaussian fitting method considering two spin−orbit doublets and two shakeup satellites. The binding energy peaks for Ni 2p3/2 and Ni 2p1/2 located at 855.6 eV and 873.4 eV, respectively (Fig. 3b), demonstrating the existence of both Ni^2+^ and Ni^3+^. The strong peaks at 779.4 eV for Co 2p3/2 and 796.5 eV for Co 2p1/2 were displayed in Fig. 3c, confirming both Co^3+^ and Co^2+^ in the GF/NiCo_2_S_4_ sample. Figure 3d showed the core-level spectrum of the S2p region, in which the binding energies at 163.6 and 161.7 eV attributed to the bond between metal and S. Besides, the binding energy at 162.8 eV assigned to S^2−^. These results showed that the chemical composition of GF/NiCo_2_S_4_ contain C, O, Co^2+^, Co^3+^, Ni^2+^, Ni^3+^ and S^2−^, which were in good agreement with the results in previous reports^29^,^30^. Figure 3e showed the XRD spectrum of NiCo_2_S_4_ that was collected from the Teflon autoclave where the GF/NiCo_2_S_4_ products were synthesized. The characteristic peaks at 16.3, 26.8, 31.6, 38.3, 50.5 and 55.3° could be respectively indexed to (111), (220), (311), (400), (511) and (440) diffractions of the cubic NiCo_2_S_4_ phase (JCPDS Card No.20-0782)^26^. No peaks from other crystallized phases could be observed from the sample, indicating the formation of pure NiCo_2_S_4_ phase, consistent with the TEM results.

The mechanical property is a key factor that needs to be considered when making wearable devices. The tensile strength of GF-801, GF-802 and GF/NiCo_2_S_4_ was measured by using a universal test tensile machine (Fig. S5). GF/NiCo_2_S_4_ had the highest tensile strength up to 226 MPa while the GF-801 and GF-802 exhibited 221 MPa and 182 MPa, respectively (Fig. 3f), comparable to those of wet-spun RGO fibers^31^. Moreover, the conductivity of GF/NiCo_2_S_4_ (133 S cm^−1^) was much larger than that of GFs (26 to 39 S cm^−1^). In addition, the fiber density of GF/NiCo_2_S_4_ (1.30 g cm^−3^) was 2 to 5 times greater than that of GFs (0.24 to 0.67 g cm^−3^) (Table 1), revealing the successful deposition of NiCo_2_S_4_ onto GFs. When compared to the recently reported Nickel coated cotton yarns (2.33 g cm^−3^), GF/NiCo_2_S_4_ were still much more lightweight.

### Three-electrode configuration tests of electrodes

**Figure 4:**
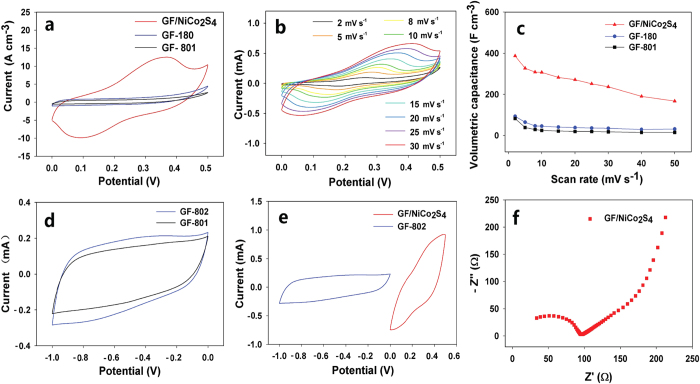
Three-electrode cell data for fibers. (**a**) CV curves of single fibers at the scan rate of 20 mV s^−1^ in aqueous 2.0 M KOH electrolyte from 0 to 0.5 V. (**b**) CV curves of GF/NiCo_2_S_4_ at scan rates from 2 to 30 mV s^−1^. (**c**) The volumetric capacitance as a function of scan rate for different fibers. (**d**) CV curves of single fibers at the scan rate of 50 mV s^−1^ in aqueous 2.0 M KOH electrolyte from −1.0 to 0 V. (**e**) Comparative CV curves obtained for the GF-802 and GF/NiCo_2_S_4_ fibers at the scan rate of 50 mV s^−1^. (**f**) Electrochemical impedance measurement of GF/NiCo_2_S_4_ in 2.0 M KOH solution.

There are two different mechanisms involved in the energy storage of supercapacitors. One is electrochemical double layer capacitance (EDLC) because the electrolyte ions are absorbed on the oppositely charged electrode surfaces. And the other is pseudocapacitance, in which fast electrochemical reactions occur at the surface of an electrochemically active material. We firstly tested the electrochemical performance of the as-prepared fibers in a three-electrode cell in a 2 M KOH solution. In order to investigate the effects of solvothermal reaction on GFs, the GFs treated following the similar procedure of GF/NiCo_2_S_4_ but without adding CoCl_2_·6H_2_O and NiCl_2_·6H_2_O were denoted as GF-180 for comparison in the three-electrode cell test. Figure 4a compared the cyclic voltammetry (CV) curves between GF-801, GF-180 and GF/NiCo_2_S_4_ at a scan rate of 20 mV s^−1^ from 0 V to 0.5 V, which revealed that the current density of GF/NiCo_2_S_4_ was several times larger than those of GF-801, GF-180. The well-defined redox peaks in the CV curve of GF/NiCo_2_S_4_ indicated the presence of pseudocapacitance in KOH. Figure 4b presented the typical CV curves of GF/NiCo_2_S_4_ with various scan rates (2–30 mV s^−1^). These distinct peaks might be attributed to the reversible Faradaic redox processes of Co^2+^/Co^3+^/Co^4+^ and Ni^2+^/Ni^3+^ based on the following reactions:^32^


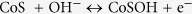



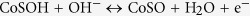



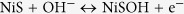


Since the redox potentials of Co^2+^/Co^3+^ and Ni^2+^/Ni^3+^ were close, the two redox peaks overlapped together^33^. The first pair of redox peaks might be assigned to the redox reaction among from NiCo_2_S_4_ to NiSOH and CoSOH. Another pair probably came from the conversion between CoSOH and CoSO^29^. Figure 4c showed that the calculated volumetric capacitance of GF/NiCo_2_S_4_ was up to 388 F cm^−3^ at a scan rate of 2 mV s^−1^, which was four times larger than that of GF-180 and nearly five times greater than that of GF-801. Even when the scan rate was up to 50 mV s^−1^, the GF/NiCo_2_S_4_ still achieved a volumetric capacitance of 167 F cm^−3^.

In an asymmetric supercapacitor, the charges stored in positive electrode and negative electrode need to be balanced to reach the best performance. Therefore, we further tested the CVs of GF-801 and GF-802 in the scan potential window from −1.0 to 0 V at a scan rate of 50 mV s^−1^ to figure out which fibers were suitable as the negative electrode to match GF/NiCo_2_S_4_ as the positive one. From Fig. 4d,e, both of the charge of GF-802 and GF/NiCo_2_S_4_ were calculated to be 3.75 mC cm^−1^ based on the equation Q = C_L_*ΔE, where Q is the charge in mC cm^−1^, C_L_ is the specific capacitance in length in mF cm^−1^, ΔE is the potential window in V. So the GF-802 would be the proper choice. The electrochemical impedance measurement showed that the GF/NiCo_2_S_4_ had a rather small equivalent series resistance about 100 Ω (Fig. 4f), which was much smaller than that of the wet-spun RGO fibers (2500 Ω) in our previous report^14^. These results confirmed that the GF/NiCo_2_S_4_ showed excellent electrochemical performances.

### Two-electrode configuration test of asymmetric supercapacitors

**Figure 5:**
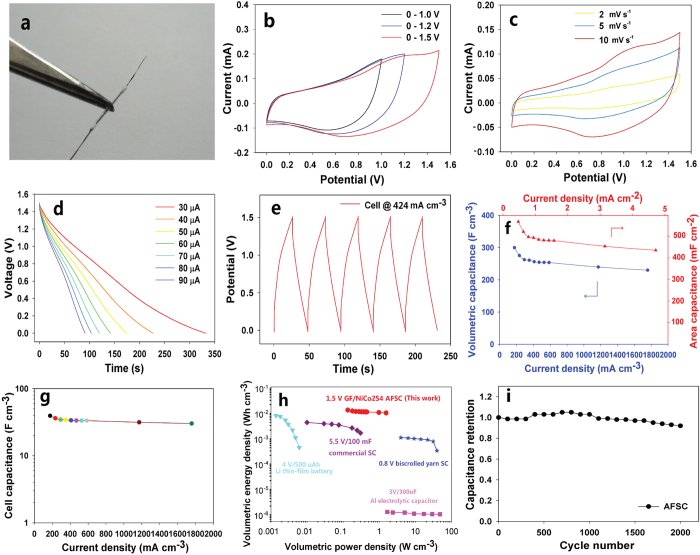
Two-electrode cell data for devices. (**a**) A digital image of a 1 cm AFSC. (**b**) CV curves of the asymmetric GF/NiCo_2_S_4_//GF-802 AFSC with different operation voltages at the scan rate of 20 mV s^−1^. (**c**) CV curves of the AFSC at scan rates from 2 to 10 mV s^−1^. (**d**) Discharged curves of AFSCs at applied currents from 30 to 90 μA. (**e**) Charged and discharged curves of AFSCs at current density of 424.3 mA cm^−3^. (**f**) Volumetric (area) capacitance vs. volumetric (area) current density for AFSCs. (**g**) Cell capacitance as a function of current density for AFSCs. (**h**) Ragone plots for our AFSCs compared with commercially available energy-storage systems. Data of the Li battery, Al electrolytic capacitors and the 5.5 V/100 mV commercial SC were reproduced from ref. 12. Data for biscrolled SC are from ref. 41. (**i**) Cycle life of the asymmetric GF/NiCo_2_S_4_//GF-802 AFSC.

We fabricated a high-voltage AFSC using the GF/NiCo_2_S_4_ as the positive electrode and GF-802 as the negative electrode, as illustrated in Fig. 5a. Figure 5b showed the CV profiles of GF/NiCo_2_S_4_// GF-802 asymmetric supercapacitors at the scan rate of 20 mV s^−1^ in different potential windows. Clearly, the asymmetric device presented rectangular-like CV curves even at a high potential window up to 1.5 V, which was nearly twice as high as those of recently reported symmetric FSCs^13^,^16^,^17^. Figure 5c exhibited its CV at different scan rates from 2 to 10 mV s^−1^. A pair of redox peaks could be seen in the CV measurement, which revealed the reversible Faradaic redox processes of NiCo_2_S_4_ in the two-electrode cell. The CV profiles could also keep rectangular-like shape even at a higher scan rate of 100 mV s ^−1^ (Fig. S6), manifesting its excellent capacitive behavior.

Figure 5d displayed the discharge curve of the AFSC at different applied current from 30 to 90 μA. Moreover, the galvanostatic charge/discharge curves of the AFSC at 424.3 mA cm^−3^ were shown in Fig. 5e. The volumetric and area capacitance of GF/NiCo_2_S_4_ at different applied current densities were calculated in Fig. 5f based on Fig. 5e. The GF/NiCo_2_S_4_ achieved a maximum volumetric capacitance and area capacitance up to 300 F cm^−3^ at 175.7 mA cm^−3^ and 568 mF cm^−2^ at 0.5 mA cm^−2^, respectively. It still retained 230 F cm^−3^ at a high volumetric current density up to 1.8 A cm^−3^ (or 435 mF cm^−2^ at a high area current density up to 4.7 mA cm^−2^). When the capacitance per length was considered, the GF/NiCo_2_S_4_ achieved the maximum specific capacitance of 13.5 mF cm^−1^ (Fig. S7) which was much larger than the previous reports (Table S1)^34^,^35^,^36^,^37^,^38^. The volumetric capacitance of the asymmetric cell (normalized to the active materials including two fibers) was 39.4 F cm^−3^ at 175.7 mA cm^−3^ and retained 30.2 F cm^−3^ at 1.8 A cm^−3^, indicating good rate capability (Fig. 5g). The columbic efficiency could achieve about 92.3% at a current density of 4.7 mA cm^−2^ for the GF/NiCo_2_S_4_// GF-802 based device. And it still could maintain 73.3% at a lower current density of 1.3 mA cm^−2^ (Fig. S8). The columbic efficiency is not 100% because of losses in charges, largely because of secondary reactions, such as the electrolysis of water or other redox reactions in the supercapacitors.

The mass of active materials is very small in FSCs, thus the volumetric power or energy densities are more reliable performance metrics for FSCs compared with gravimetric power or energy densities^39^. The Ragone plots (Fig. 5h) compared the volumetric performance of the AFSCs in this work with those of commercially available energy-storage devices. The AFSCs had a maximum volumetric energy density (normalized to the active electrode) of ~12.3 mWh cm^−3^, which was about 12 folds higher than those of typical bulk SCs (5.5 V/100 mF, <1 mWh cm^−3^) and even larger than that of the 4 V/500 μAh thin-film lithium battery (0.3–10 mWh/cm^−3^). The energy density value calculated based on the active electrodes was much higher than most reported carbon-based FSCs including SWCNT@C FSCs^13^ (1.6 mWh cm^−3^), MoS_2_-rGO/MWCNT FSCs^15^ (<2.0 mWh cm^−3^), PPY@MnO_2_@RGO yarn-based FSCs^17^ (6.0 mWhcm^−3^), GCF/MnO_2_ FSCs^40^ (9.0 mWh cm^−3^), and PEDOT/MWNT biscrolled yarns FSCs^41^ (1.4 mWh cm^−3^). Even when the whole device including two fibers, separator and electrolyte all inside the polytetrafluoroethylene (PTFE) tube was considered, the stack energy density could reach up to 3.0 mWh cm^−3^, which was among the best reported values (TableS2)^12^,^13^,^14^,^15^,^16^,^17^,^40^,^41^,^42^,^43^. Figure 5i showed that the AFSC retains 92% of its initial capacity after 2,000 charge-discharge cycles under the current density of 0.4 A cm^−3^, indicating a good cycle life.

### Wearable applications of AFSCs

**Figure 6:**
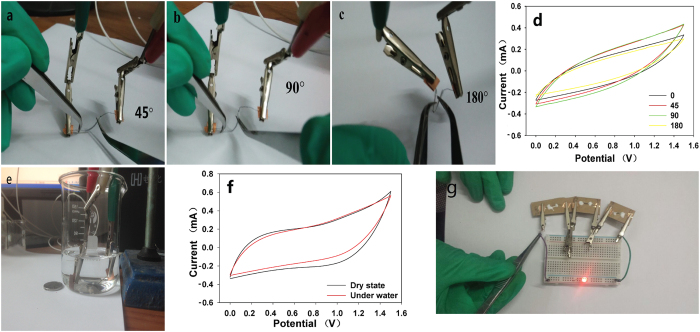
(**a**–**c**) Digital images for the AFSC at different bending angles from 45° to 180°. (**d**) CV curves of the AFSC at corresponding bending angles at scan rate of 200 mV s^−1^. (**e**) The AFSC tested under water. (**f**) CV curves of the AFSC tested in air and under water at scan rate of 100 mV s^−1^. (**g**) Three AFSCs connected in series to power a LED.

To explore the application potentials of the devices, the flexibility of the AFSCs was tested. Figure 6a–c displayed the photos of AFSCs under different bending angles (45, 90 and 180°). Figure 6d showed the CV curves of the AFSCs under the corresponding angles. The CV curves showed similar capacitive behavior with minor capacitance changes, demonstrating excellent flexibility. The AFSCs also showed impressive waterproof characteristic under water because water could not penetrated into the devices after the electrodes were packed into the PTFE tubes and sealed up by silica gel. The CV curves shown in Fig. 6f revealed that there was nearly no decrease of capacitance for AFSCs tested under water (Fig. 6e) when compared to that in atmosphere. Finally, we used three AFSCs (1 cm in length for each) connected in series to power a LED easily (Fig. 6g).

## Discussion

In summary, we have demonstrated a high-performance wearable AFSC device which shows high cell voltage (1.5 V), high volumetric capacitance (388 F cm^−3^) and high energy density (12.3 mWh cm^−3^). We discuss the detailed reasons as followings.

Firstly, the resultant GFs were lightweight (0.24 g cm^−3^), highly conductive (39 S cm^−1^), and mechanically robust (221 MPa). The solution processed reduction method provided potentials of the widespread application of GFs in different fields.

Secondly, we confirmed that the GFs could be further coated by NiCo_2_S_4_ via a solvothermal reaction. The GF/NiCo_2_S_4_ also displayed good tensile strength of 226 MPa, low density of 1.30 g cm^−3^ and high conductivity of 133 S cm^−1^. It is shown that the GF/NiCo_2_S_4_ possesses a specific volumetric capacitance up to 388 F cm^−3^ at a scan rate of 2 mV s^−1^ in a three-electrode cell or 300 F cm^−3^ at 175.7 mA cm^−3^ and 568 mF cm^−2^ at 0.5 mA cm^−2^ in a two-electrode cell. So it is reasonable to speculate that other active materials could probably be deposited on GFs. This study provides a way to construct high cell voltage supercapacitors in aqueous electrolyte.

Thirdly, the AFSCs based on GF/NiCo_2_S_4_ exhibit remarkable volumetric energy density (12.3 mWh cm^−3^) which is larger than that of thin-film lithium batteries. Good life cycle stability (92% device capacitance retention over 2000 cycles) could also be achieved. An AFSC could be charged to 1.5 V and three units in series could be used to power a LED easily. The AFSCs also exhibited excellent flexibility and are robust enough in wearable textiles.

Therefore, the AFSCs are promising wearable energy storage devices for the next generation flexible and wearable electronics.

## Methods

### Preparation of graphene fiber electrodes

Graphene oxide (GO) was synthesized by using a modified Hummers method as reported in the previous report^44^. The GFs were fabricated according to our reported method^45^. Typically, a mixture of 10 ml GO suspension (2.0 mg ml^−1^) and 100 mg AA was stirred for 60 minutes (min) and further injected into silicone tubing with an inner diameter of 3.0 mm. Then the silicone tubing were sealed by spring-water-stoppers and used as reactors at 80 °C in a water bath for 1 hour (h). After the reaction, the wet GFs inside the silicone tubing were obtained. The dry GFs were obtained after the wet GFs were drying in air at 100 °C for 2 h. In order to get rid of the residual AA, the GFs were washed by DI water and further dried in air at 100 °C for 2 h again (Fig. S9). The GFs made from 2.0 mg ml^−1^ and 2.5 mg ml^−1^ GO solution were denoted as GF-801 and GF-802, respectively.

### Preparation of GF/NiCo_2_S_4_ electrodes

The GF/NiCo_2_S_4_ was synthesized based on GF-801 as following: 1.0 mmol CoCl_2_·6H_2_O and 0.5 mmol NiCl_2_·6H_2_O were dissolved into a 20 mL ethylene glycol solution. The mixture was stirred for 30 min to form a transparent pink solution. Afterwards, 3.0 mmol thiourea was added into the stirring pink solution. The stirring was keeping for another 30 min. The solution and 20 cm GF-801 were then transferred to a Teflon-lined stainless steel autoclave and kept at 180 °C for 24 h. After the solvothermal reactions, the GFs were successfully coated by NiCo_2_S_4_ nanospheres. The products (GF/NiCo_2_S_4_) were carefully rinsed several times with de-ionized water and absolute ethanol, and finally dried in air. The GFs treated following the similar procedure without adding CoCl_2_·6H_2_O and NiCl_2_·6H_2_O were denoted as GF-180 for comparison in the three-electrode cell test.

### Preparation of PVA-KOH electrolyte

PVA-KOH gel was used both as a solid electrolyte and separator. PVA-KOH was simply made as following: 200 mg of PVA powder was added to 2.0 mL of DI water. The mixture was heated to 85 °C under vigorously stirring until the solution became clear. Afterwards, 2 ml of KOH solution (2.0 M) was added into the PVA gel to make the PVA-KOH.

### Assembly of fiber SCs

The GFs and GF/NiCo_2_S_4_ were dipped into the PVA-KOH for 5 min and after the gel was solidified, they were packaged into a PTFE tube with a diameter of 0.3 mm. 2.0 M of KOH solution was further injected into the PTFE tube. Finally, the two ends of PTFE tube was sealed up by silica gel. The GFs were used as negative electrodes and the GF/NiCo_2_S_4_ were positive electrodes in asymmetric supercapacitors.

### Characterization

Scanning electron microscopy (SEM) images were recorded on Merlin with an accelerating voltage of 20.0 kV. The transmission electron microscopy (TEM) was conducted on JEM-2100F. X-ray diffraction (XRD) patterns were collected from a Bruker AXS D8-Advanced diffractometer with Cu Kα radiation (λ = 1.5418 Å). X-ray photoelectron spectroscopy (XPS) was conducted on ESCALAB 250Xi, Thermo Scientific. Tensile strength tests were conducted on Wance Testing Machine. All electrochemical measurements were performed on CHI660E electrochemical workstation (Chenhua, China). For example, a three-electrode system was carried out with a GF/ NiCo_2_S_4_ as a working electrode with the length of 9 mm, an Ag|AgCl (3.0 M KCl) reference electrode and a platinum wire counter electrode.

## Additional Information

**How to cite this article**: Cai, W. *et al.* Transition metal sulfides grown on graphene fibers for wearable asymmetric supercapacitors with high volumetric capacitance and high energy density. *Sci. Rep.* **6**, 26890; doi: 10.1038/srep26890 (2016).

## Supplementary Material

Supplementary Information

Supplementary Video

## Figures and Tables

**Table 1:** Physical properties of fibers in this work.

Fibers	Diameter(μm)	Tensile Strength(MPa)	Density(g cm^−3^)	Conductivity(S cm^−1^)
GF-801	118	221	0.24	39
GF-802	127	182	0.67	26
GF/NiCo_2_S_4_	76	226	1.30	133
