# Supplementary material for: Transition metal sulfides grown on graphene fibers for wearable asymmetric supercapacitors with high volumetric capacitance and high energy density
Source: Sci Rep. 2016 Jun 1;6:26890. doi: 10.1038/srep26890 (PMC4888745; doi:10.1038/srep26890)
Supplement: Supplementary Information [file srep26890-s1.doc]

**Supplementary Information**

**Transition metal sulfides grown on graphene fibers for wearable asymmetric supercapacitors with high volumetric capacitance and high energy density**

Weihua Cai ^a^, Ting Lai ^a^, Jianwei Lai ^a^, Haoting Xie ^a^, Liuzhang Ouyang ^b^, Jianshan Ye ^a，*^ and Chengzhong Yu ^c，*^

^a^ College of Chemistry and Chemical Engineering, South China University of Technology, Guangzhou 510641, People's Republic of China.

^b^ College of Materials Science and Engineering, South China University of Technology, Guangzhou, 510641, People's Republic of China.

^c^ Australian Institute for Bioengineering and Nanotechnology, The University of Queensland, Brisbane, Australia

^*^ Corresponding author. E-mail: jsye@scut.edu.cn; c.yu@uq.edu.au

**List of Contents**

1. **A video**

**II. Supplementary Calculation Methods**

**III. Supplementary Figures**

1. **A video**

A video demonstrated that two devices (1 cm in length for each) connected in series could be used to power a LED.

**II. Supplementary Calculation Methods**

(1) For a three-electrode cell calculation:

A three-electrode configuration consisting of an Ag/AgCl electrode as the reference electrode, a platinum wire as the counter electrode, and a single fiber as the working electrode in 2.0 M KOH electrolyte was used for the capacitance measurements on a workstation (CHI 660E). The specific volumetric capacitance (C_v_) of fibers was calculated from their CV curves according to the following equation: C_v_= C_Electrode_ / V_fiber_, where V_fiber_ is the volume of the working electrode.

The fiber is considered as a cylinder to calculate its volume.

V_fiber_ = 3.14*R^2^ *L_fiber_, where R is the radius of the fiber and L_fiber_ is the length that was dipped into the electrolyte.

C_Electrode_ was calculated by using the voltammetric charge integrated from CV curves according to the following formula:

C_Electrode_=$\frac{Q}{2V}$ =$\frac{1}{2Vv}\int_{V-}^{V+} i(V)dV$

where Q is the total voltammetric charge obtained by integrating the positive and negative sweeps (i(V) is the current) of a CV curve, v is the scan rate, V (V= V^+^-V^-^) represents the scanned potential window of 0.5 V in this work.

(2) For a two-electrode cell calculation:

The capacitance of the AFSC cell derived from galvanostatic discharge curves was calculated based on the following equation: C_cell_ =I·Δt/Δ*E*, where I is the discharge current, Δt is the time for a full discharge and Δ*E* represents the potential change after a full discharge.

The specific capacitance of the active material in one single electrode was calculated based on the following equation: C_single_ =2 C_cell_ / X, where X is the length (cm), area (cm^2^) or volume (cm^3^) of one single fiber electrode.

The volumetric capacitance of AFSC was calculated by C_v_=C_cell_/V, where V is the volume of two fibers without separators and electrolyte, which is about 1.7* 10^-4^ cm^-3^.

The stack capacitance of AFSC was based on C_stack_=C_cell_/V’, where V’ is the volume of two fibers, separators and electrolyte, in other words, V’ is equal to the inner volume of PTFE tube, which is about 7.07 * 10^-4^ cm^-3^.

The volumetric or stack energy density (E_x_) and power density (P_x_) of one device depicted in the Ragone plots were calculated by using the equations:

E_x_ = (1/2) ×C_x_·Δ*E*^2^

P_x_=E_v_×3600/Δt

where _X_ represents the volume or stack, Δ*E* is the potential change after a full discharge, and Δt is the time for a full discharge.

**III. Supplementary Figures**


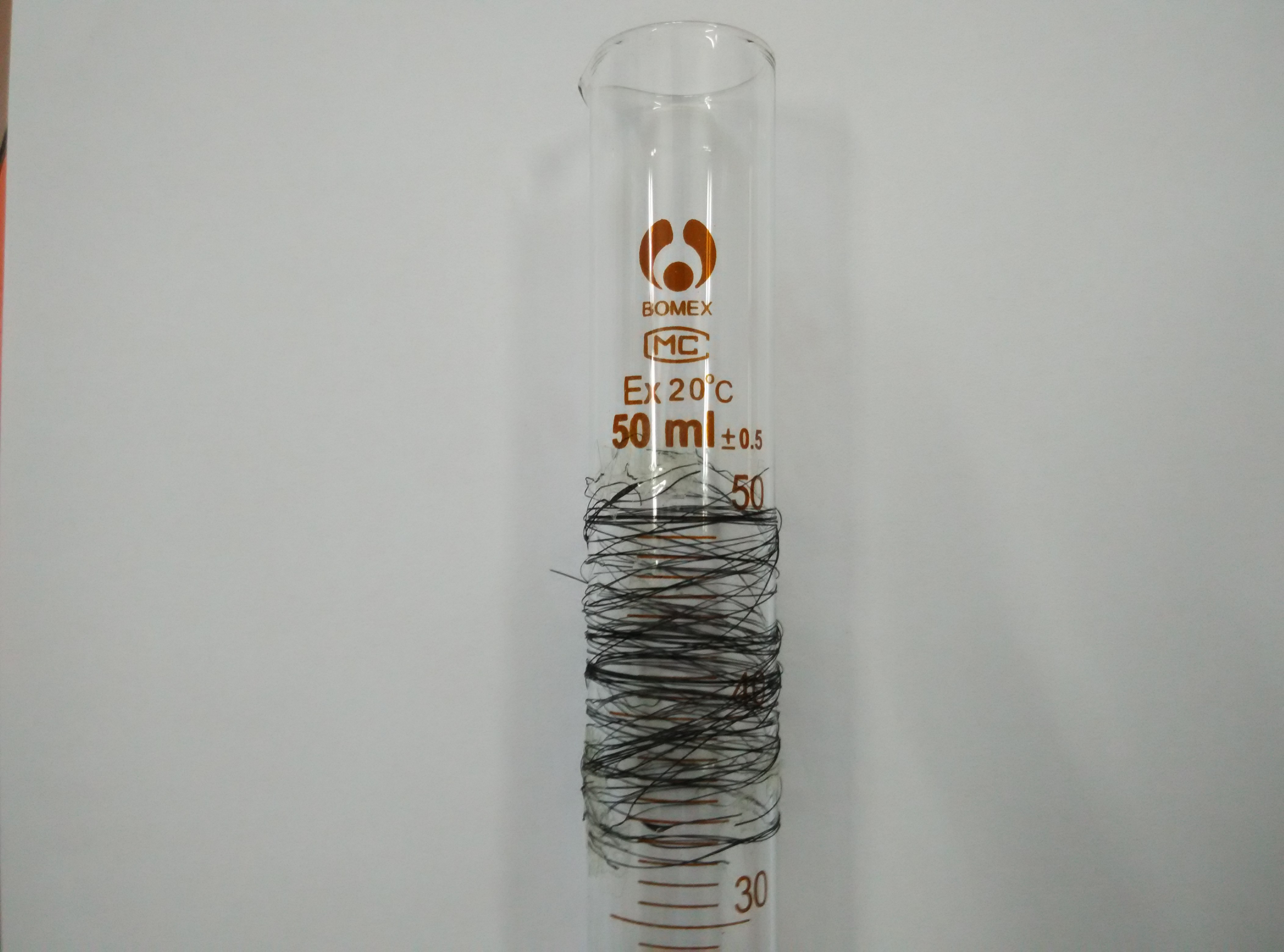

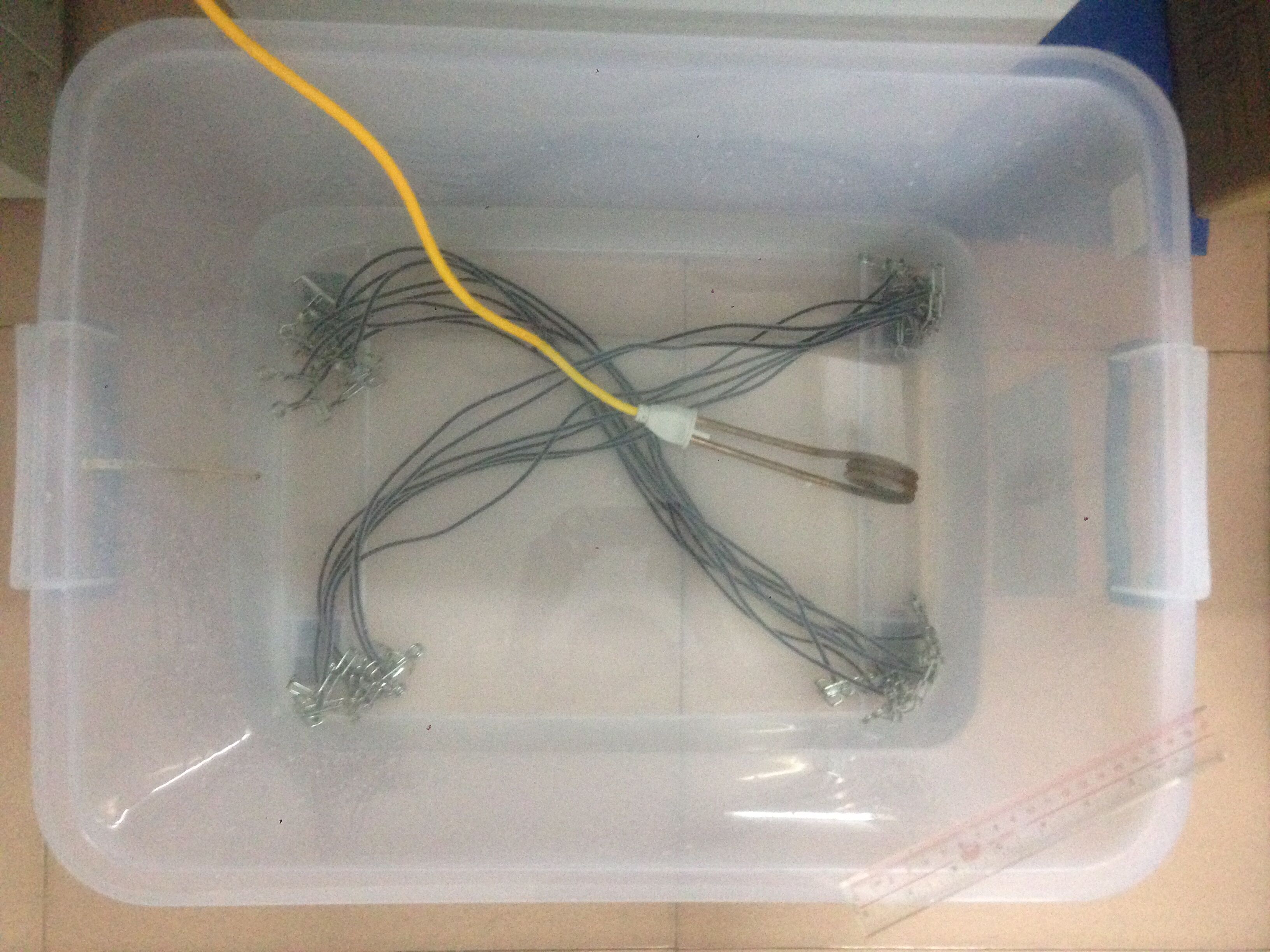

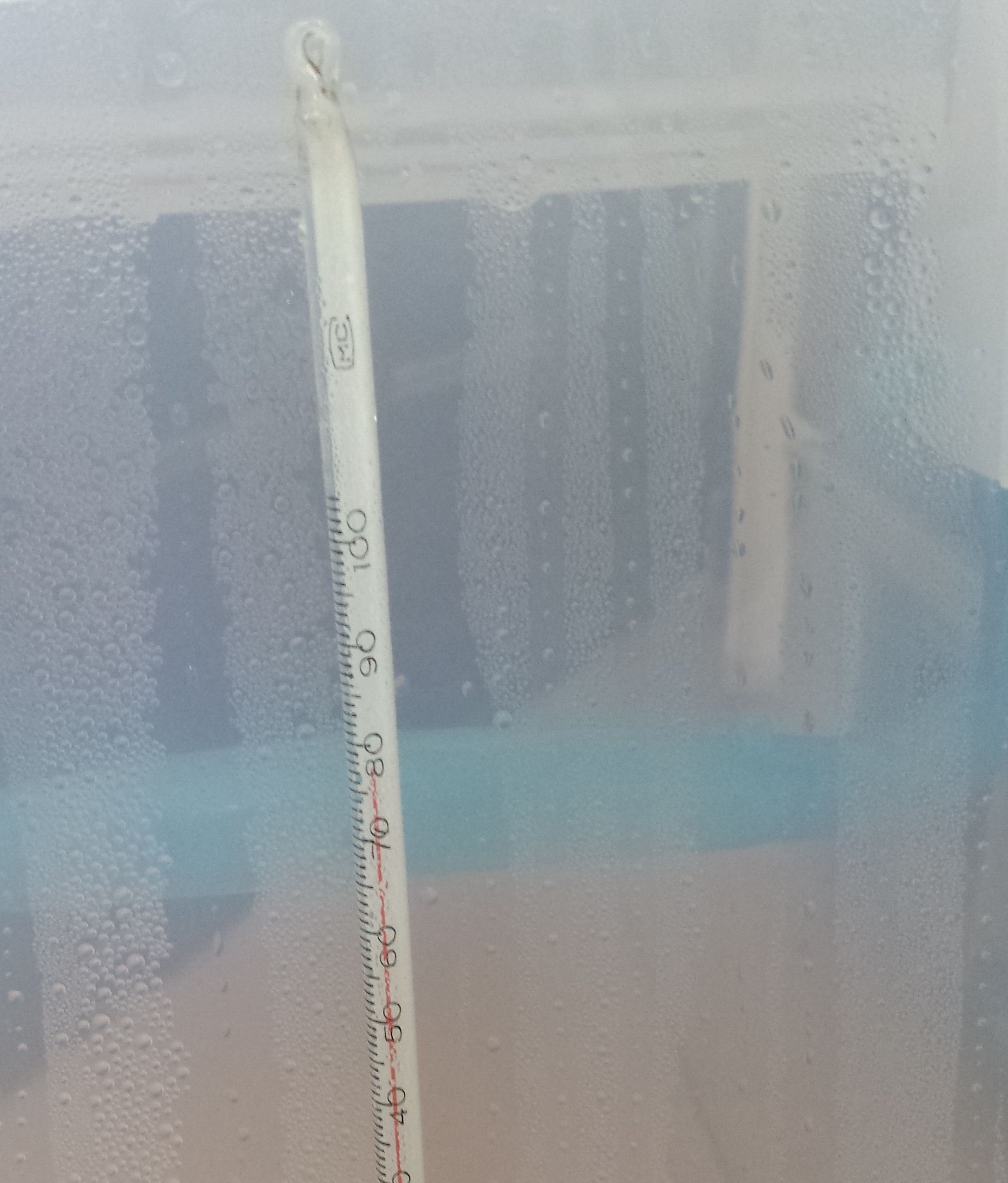


**Figure S1. Scalable fabrication of graphene fibers for more than 10 meters (1 m per segment, 10 segments in total).**


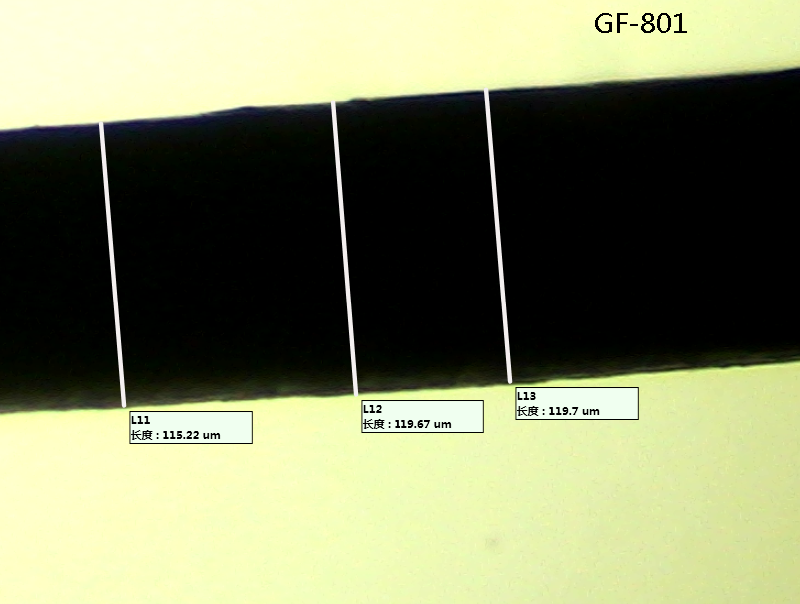

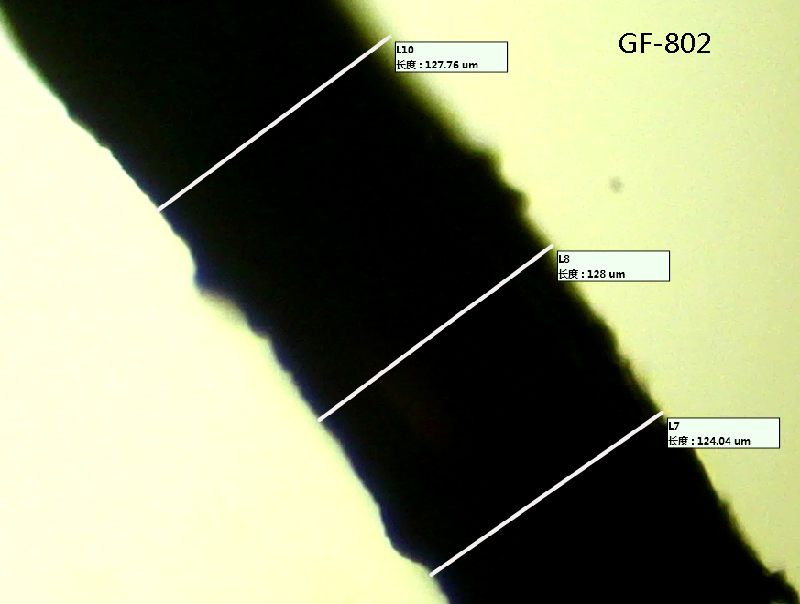


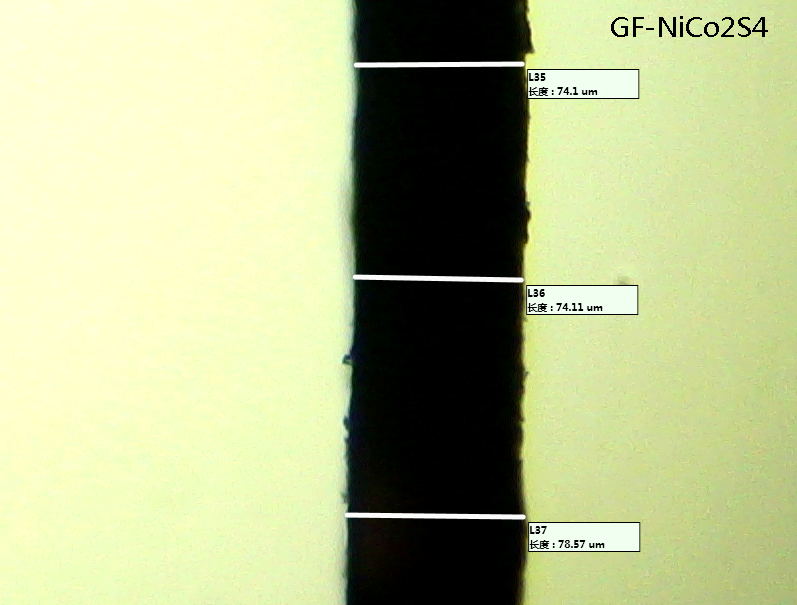


**Figure S2. Optical images of fibers for the measurements of the diameters.**

The average diameters of GF-801, GF-802 and GF/NiCo_2_S_4_ are about 118 µm, 127µm and 76 µm, respectively from Figure S2.


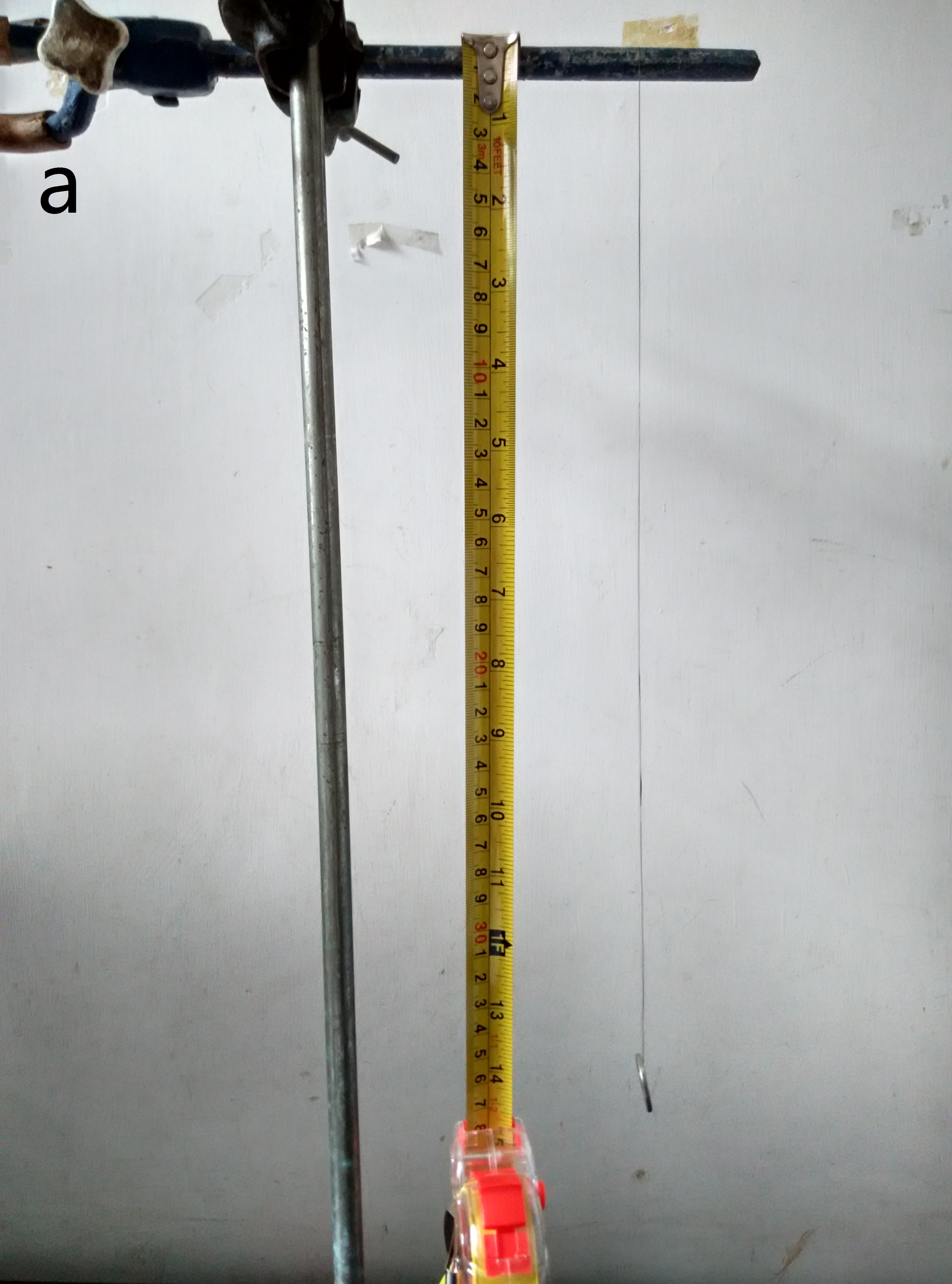

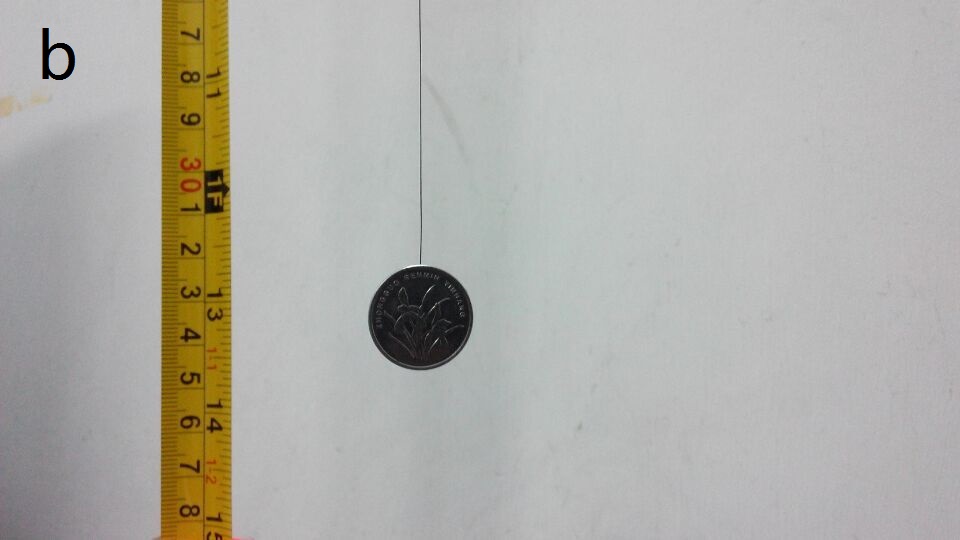


**Figure S3. A graphene fiber (30 cm) hanging a Chinese coin.**


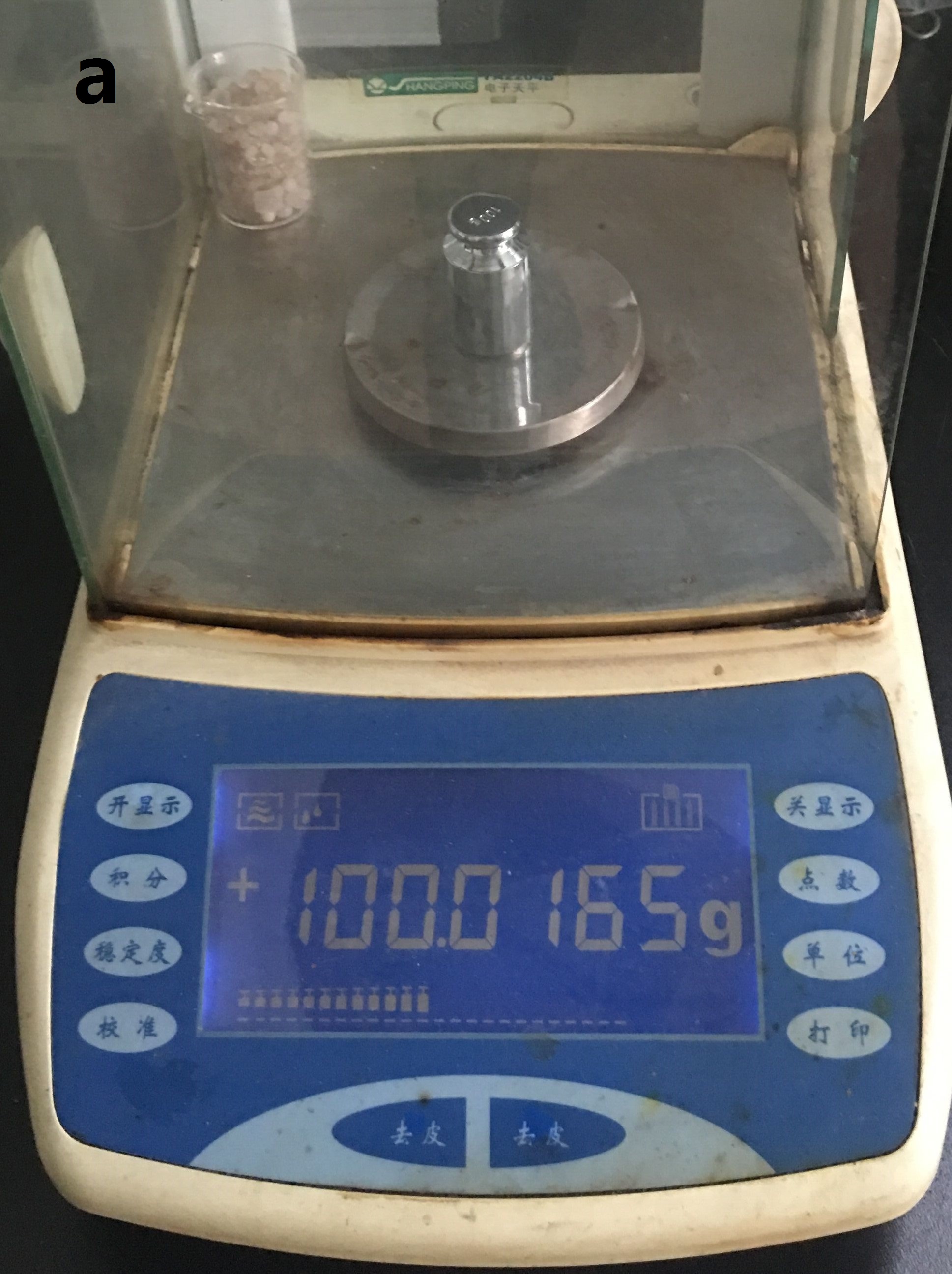

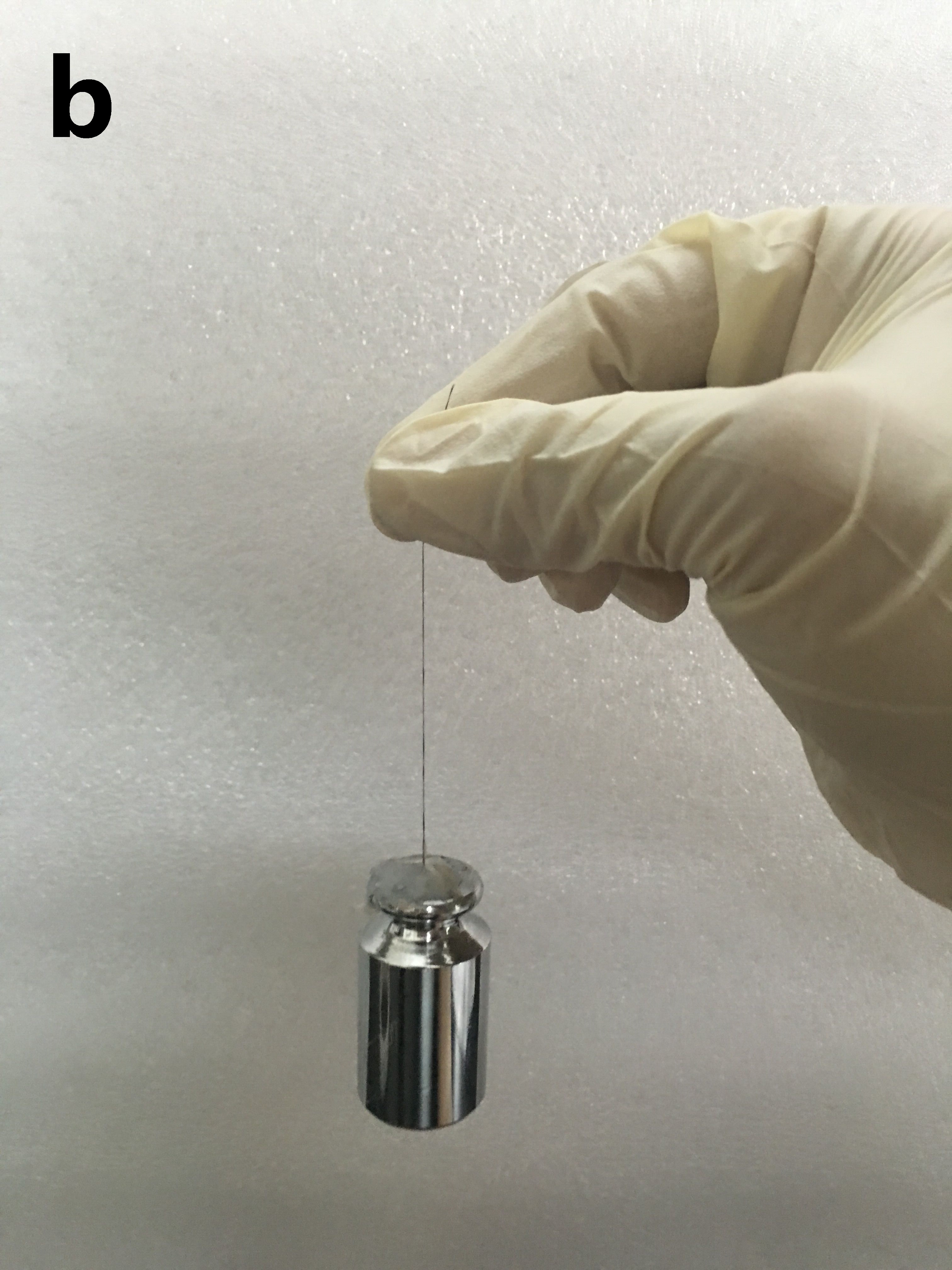

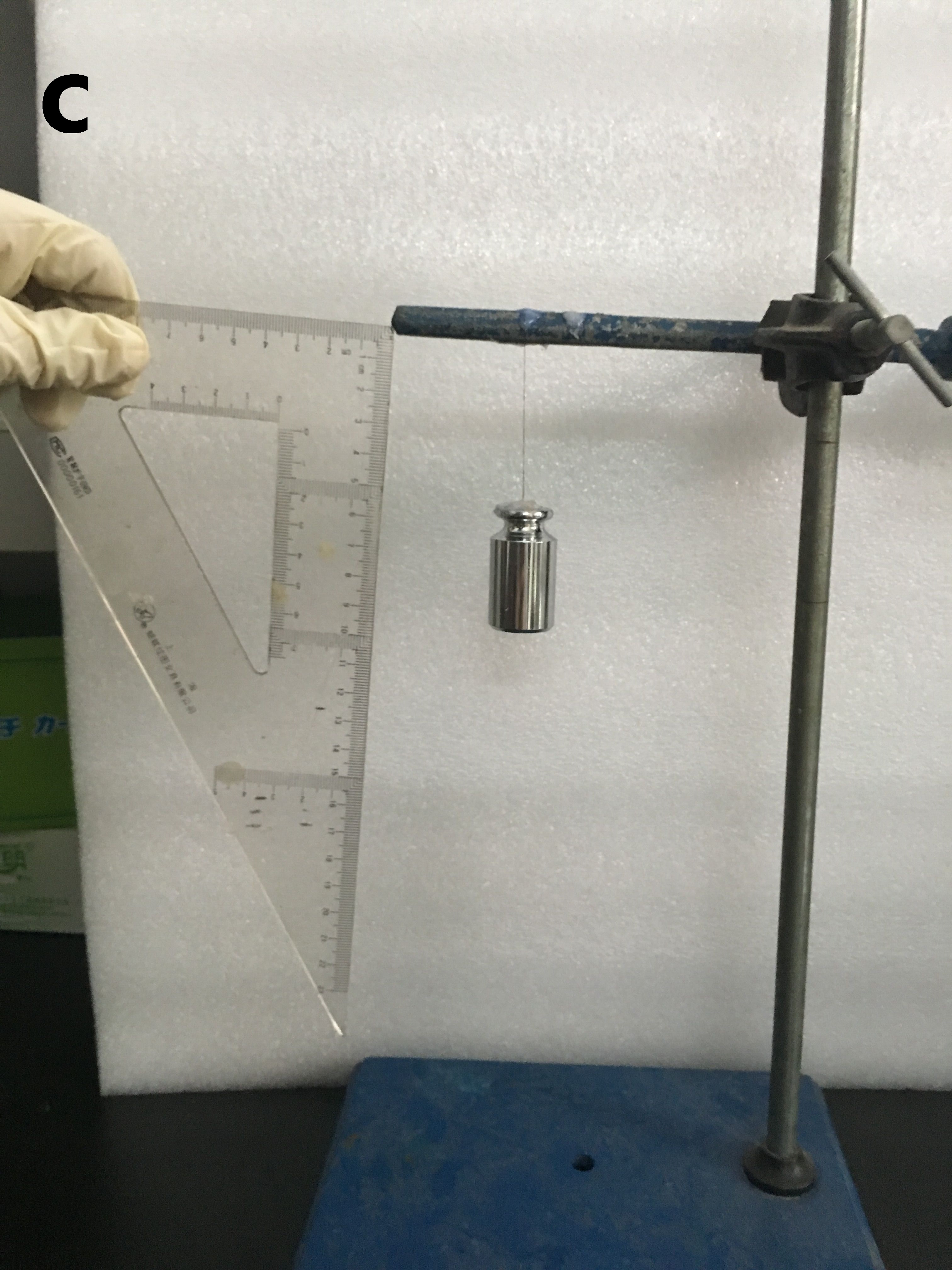


**Figure S4. A GF/NiCo_2_S_4_ (5 cm) hanging a balance weight (100g).**


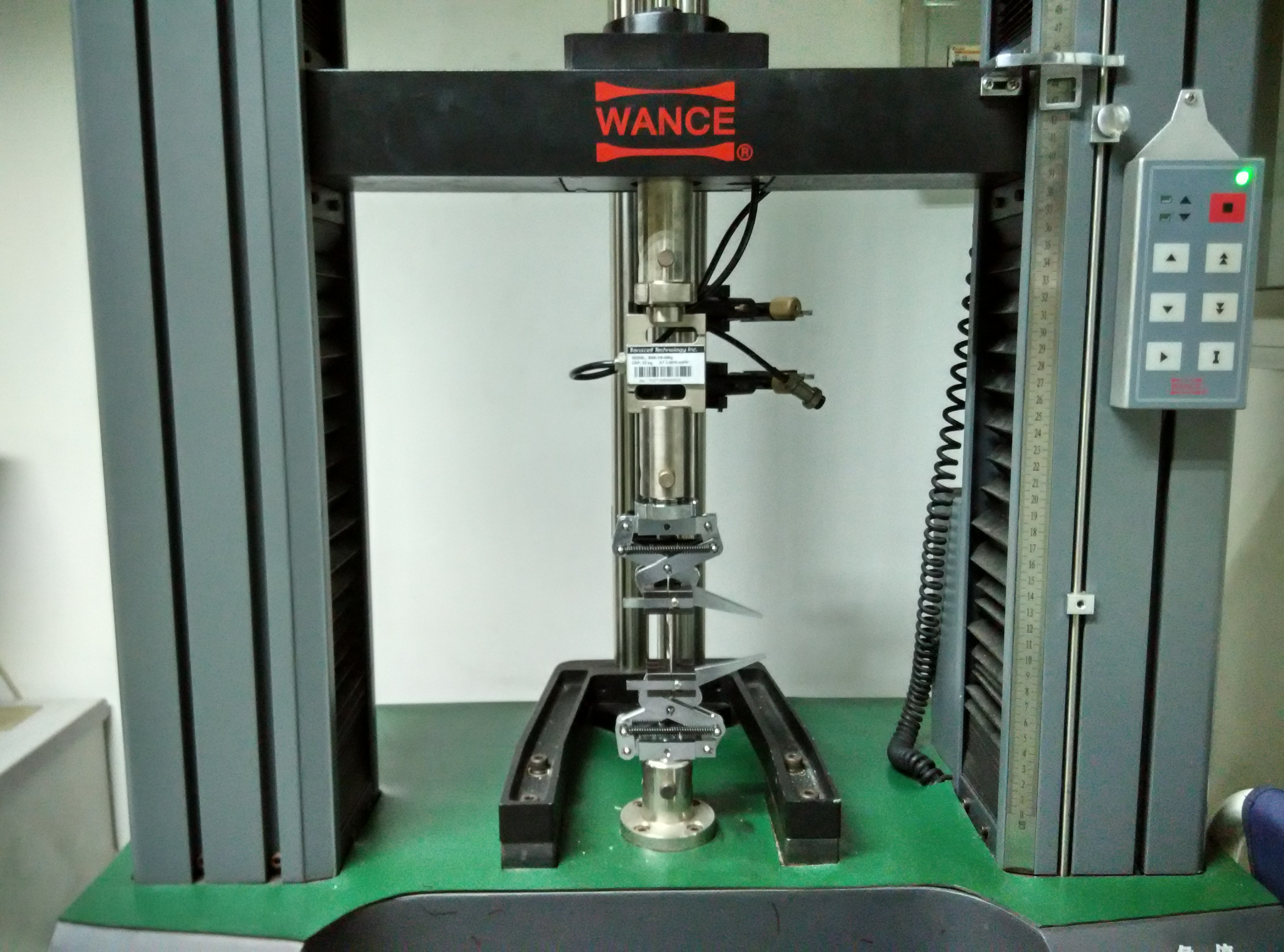

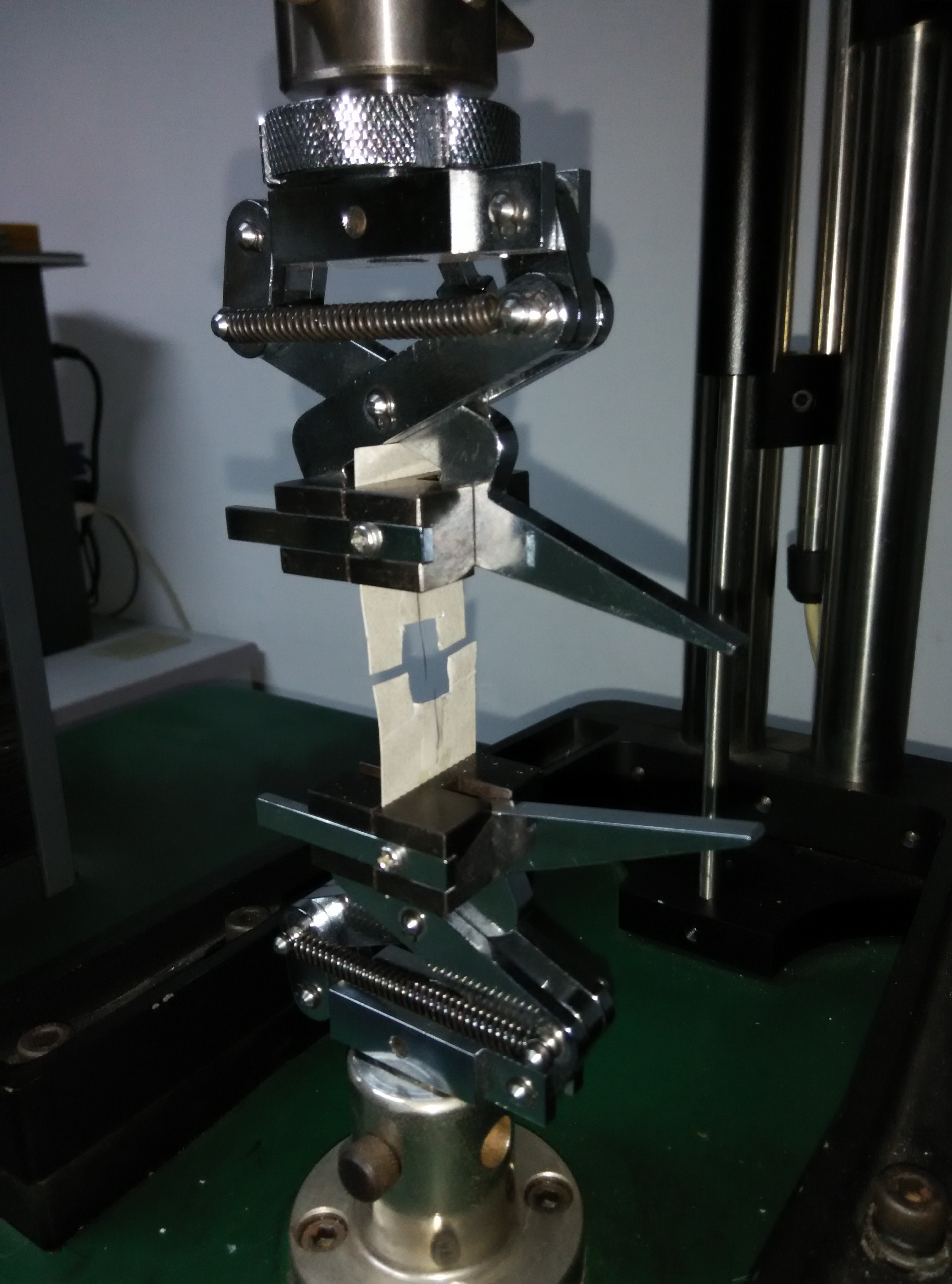

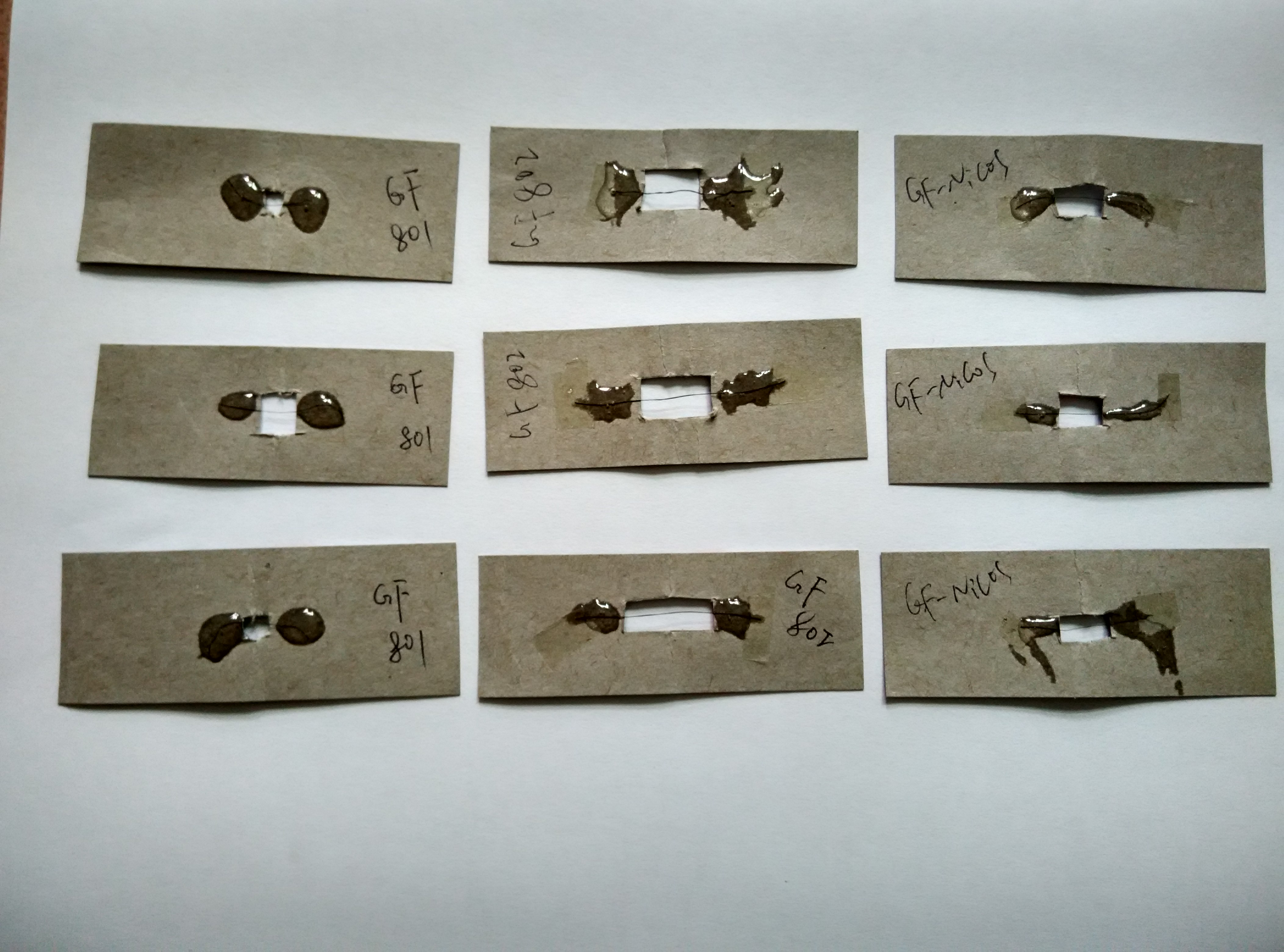


**Figure S5. Tensile strength test of fibers.**


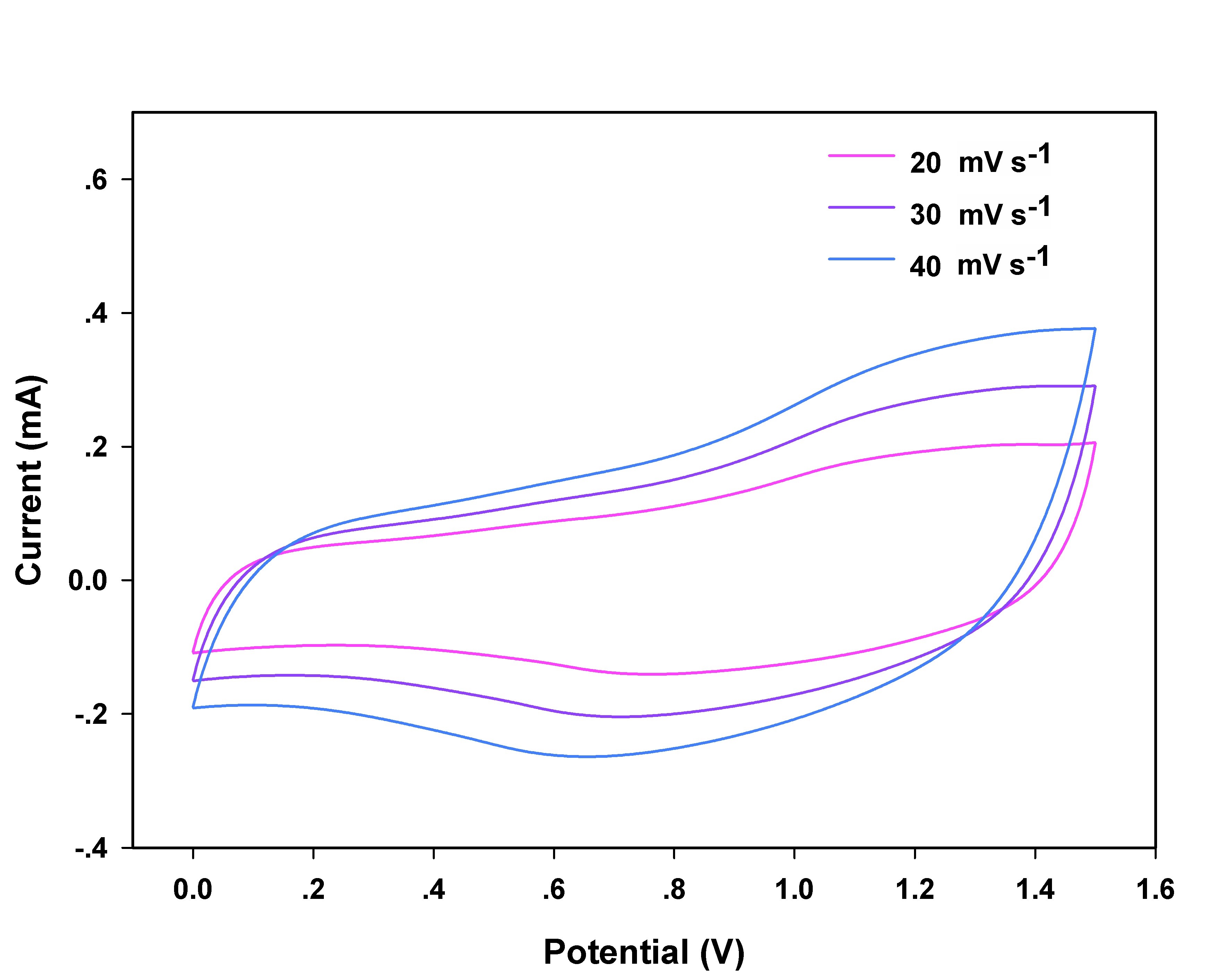

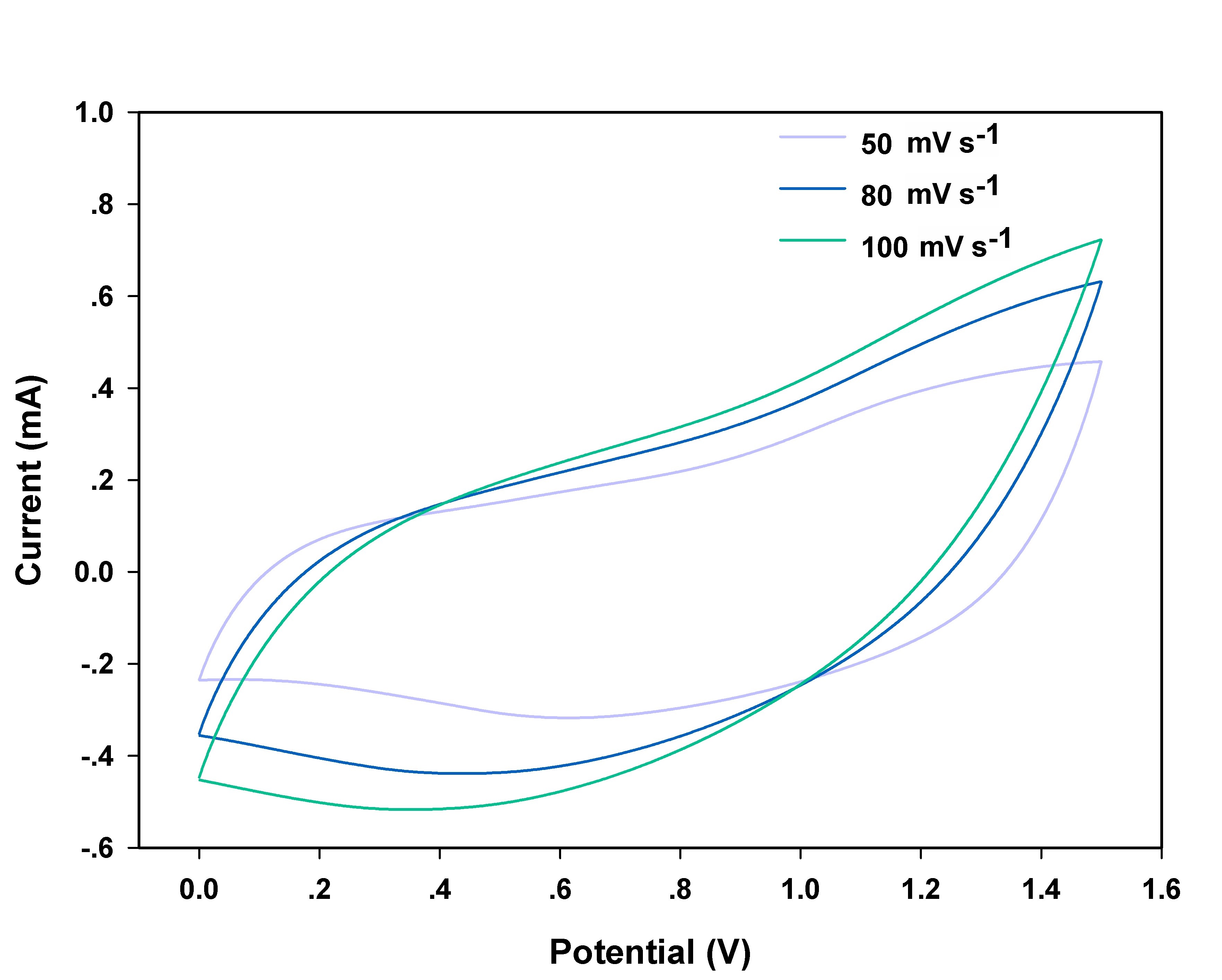


**Figure S6. CV curves of AFSCs at different scan rates from 20 to 100 mV s^-1^.**

**
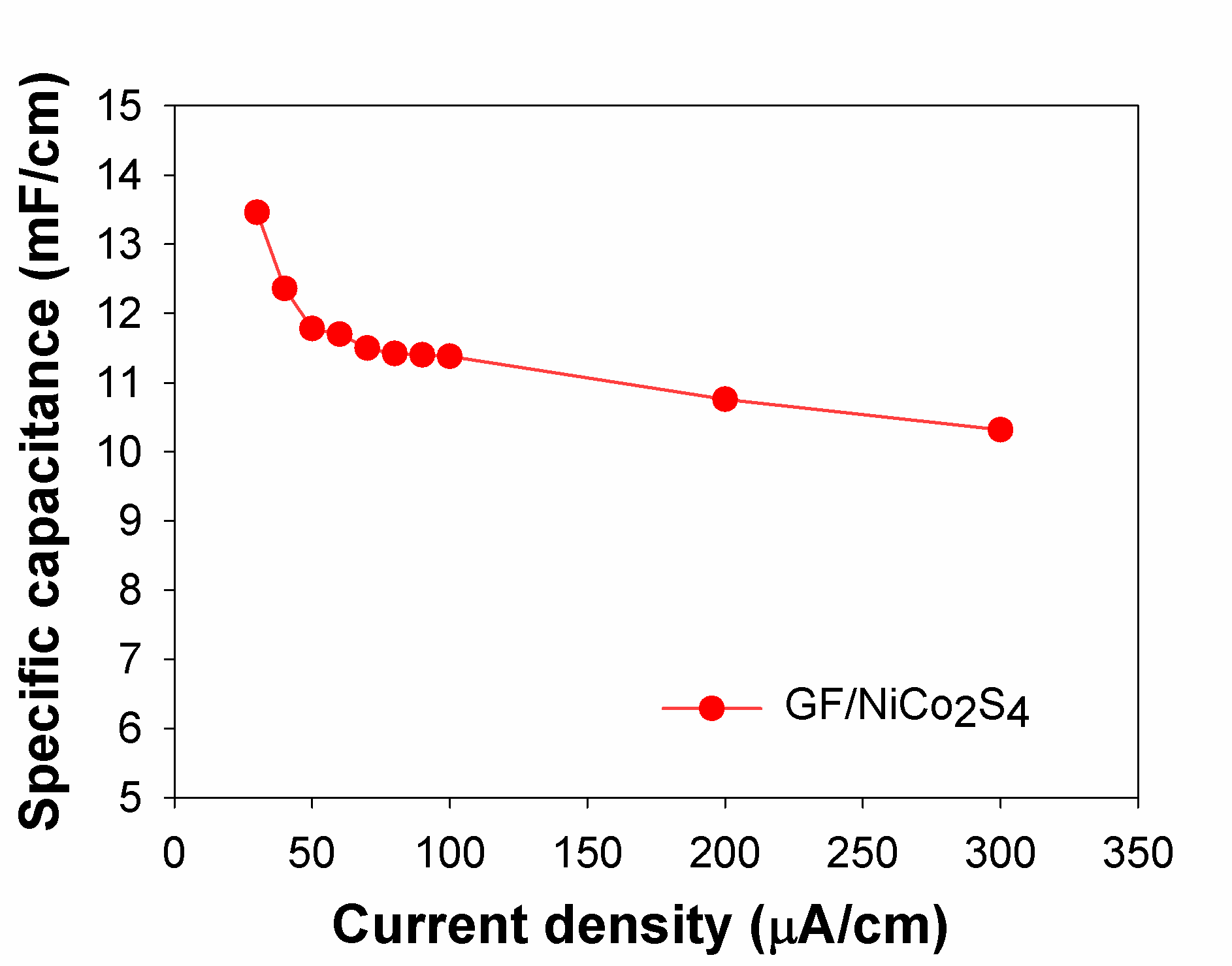
**

**Figure S7. Capacitance per length vs. current density based on two-electrode cell data.**


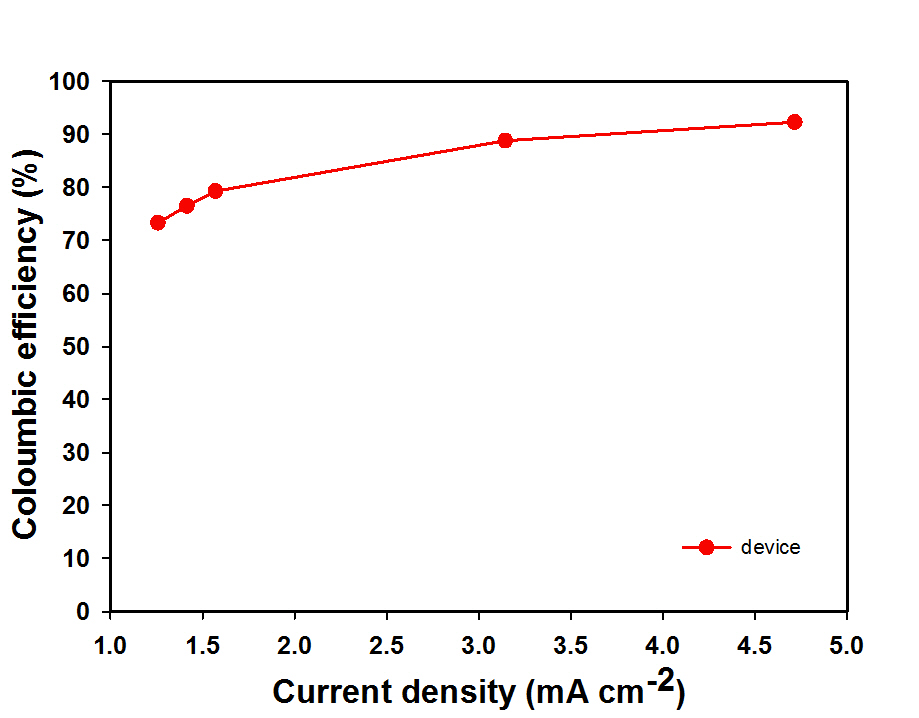


**Figure S8. Columbic efficiency of the asymmetric fiber supercapacitor at various constant current densities.**

**
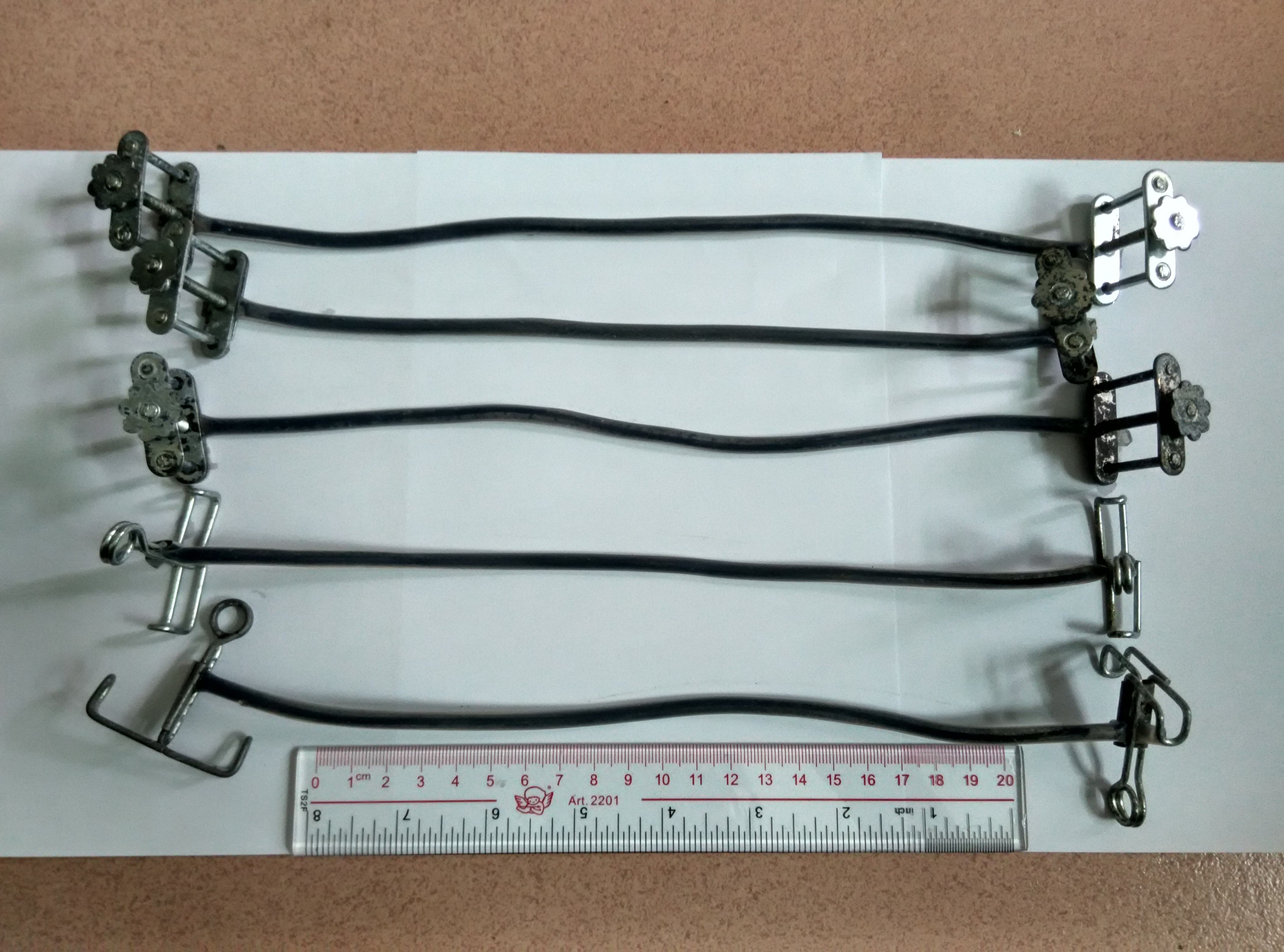

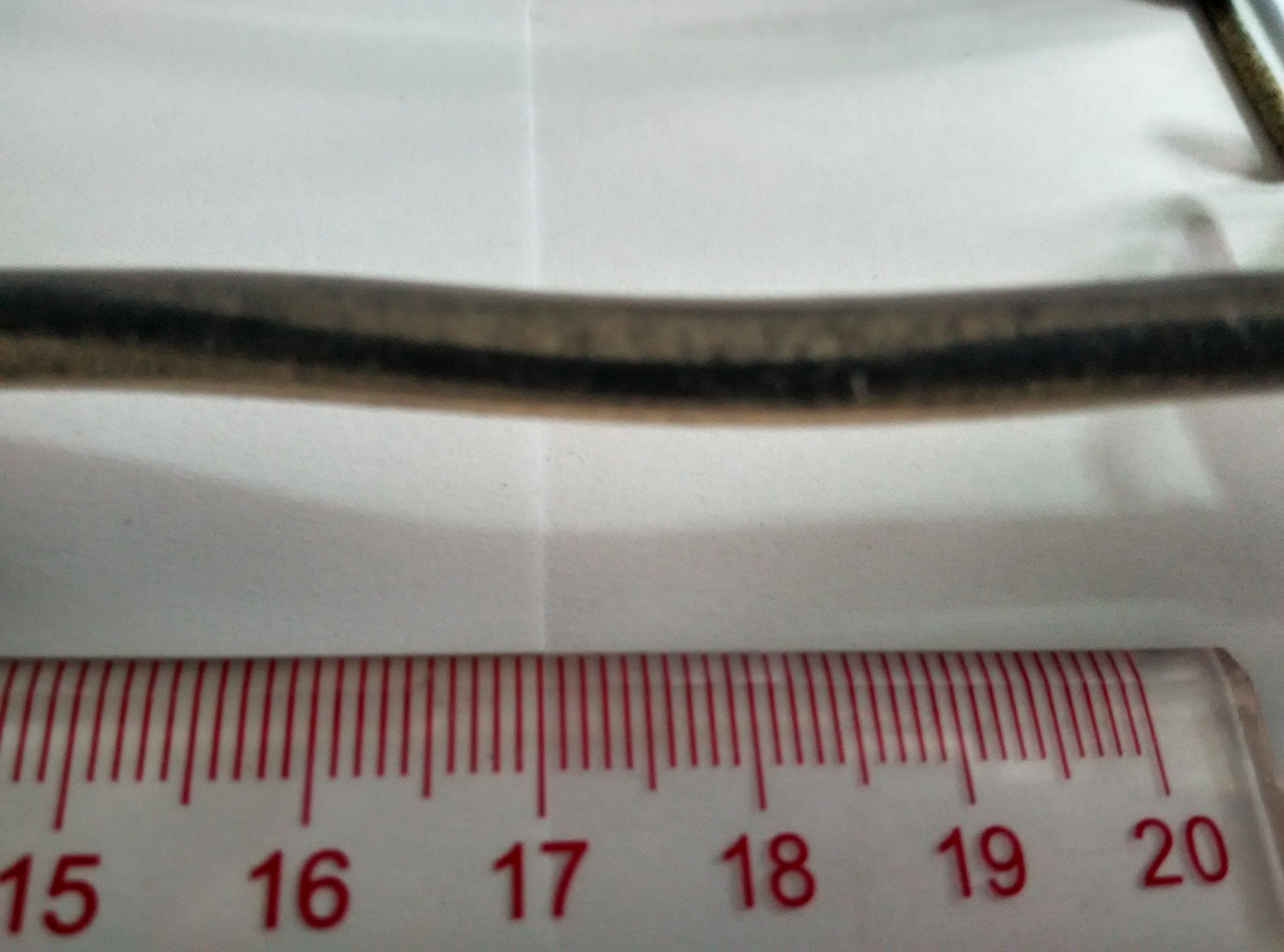

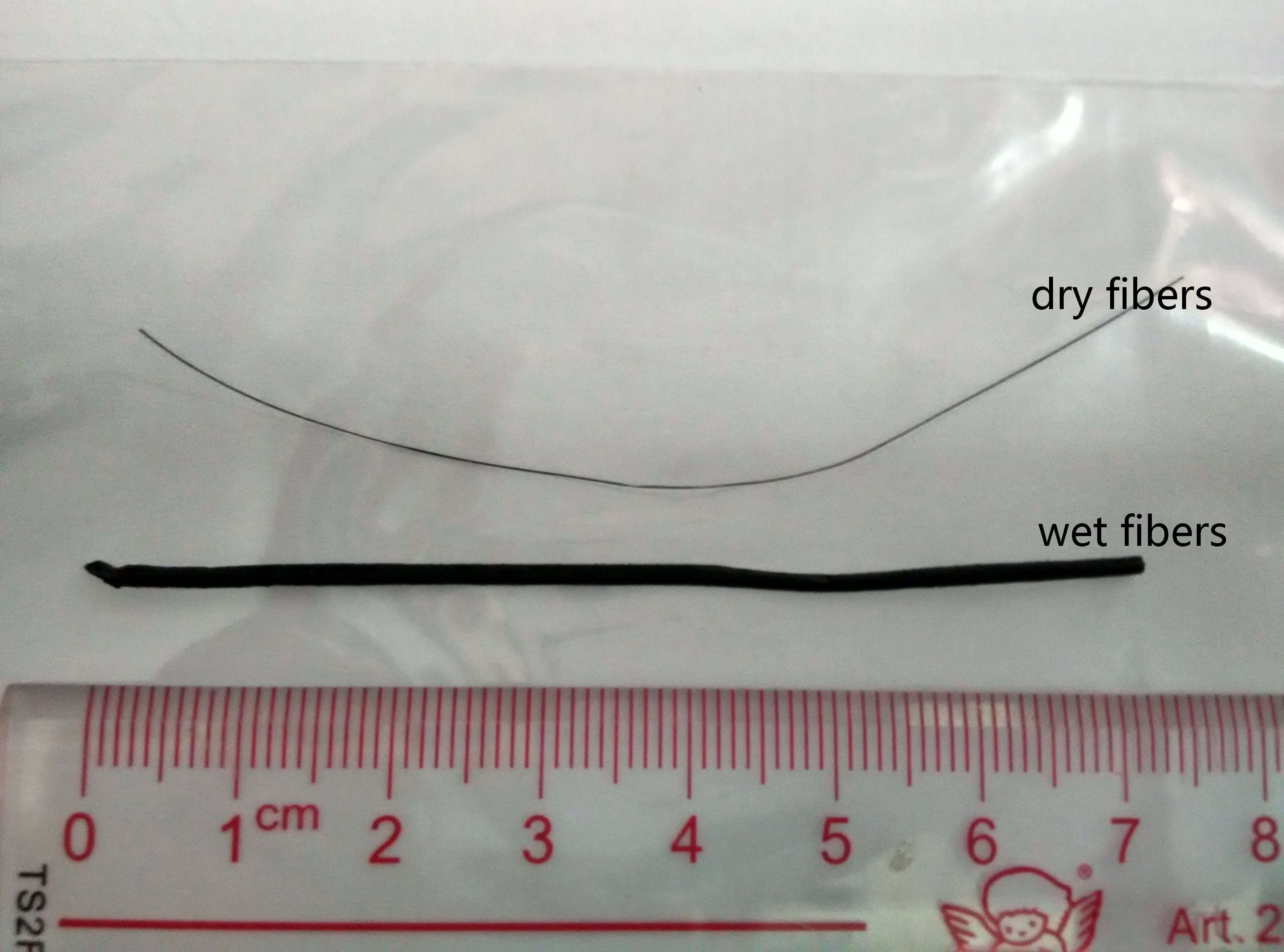
**

**Figure S9. Digital images of the fabricated process of graphene fibers (GF).**

The silicone tubing is about 25 cm in length in Figure S9.

**Table S1 Comparison of fiber supercapacitors (not planar devices)**

| **Electrode Materials** | **C_single_ (mFcm^-1^)** | **C_single_ (mFcm^-2^)** | **Csingle (Fcm^-3^)** | **Ref.** |
| --- | --- | --- | --- | --- |
| GF/NiCo2S4 | 13.5 | 568 | 300 | This work |
| RGO+CNT | 5.3 | 177 | 158 | 34 |
| PEDOT/CNT | 0.47 | 73 | 179 | 35 |
| CNT/MnO_2_ | 0.015 | 3.01 | N | 36 |
| ZnO nanowires/MnO_2_ | 0.04 | 2.4 | N | 37 |
| ZnO nanowires/graphene | 0.025 | 2 | N | 38 |

**Table S2 Electrochemical performance of recently reported fiber supercapacitors based on two-electrode cell data.**

| **Electrode Materials** | **Voltage(V)** | **C_single_(Fcm^-3^)** | **C_v_ (Fcm^-3^)** | **E_v_(mWhcm^-3^)** | **P_v_(Wcm^-3)^** | **Wearable ?** | **Ref.** |
| --- | --- | --- | --- | --- | --- | --- | --- |
| **GF/NiCo_2_S_4_** | **1.5** | **300.0** | **39.4** | **12.3** | **1.6** | **Yes** | **This work** |
|  |  |  | **9.5(stack)** | **3.0(stack)** | **0.3(stack)** |  |  |
| **N doped CNT-graphene fibers** | **1.0** | **300.0** | **45.0(stack)** | **6.3(stack)** | **1.1** | **No** | **12** |
| **SWCNT@C** | **0.8** | **48.5** | **N** | **1.6** | **3.8** | **No** | **13** |
| **Porous graphene fibers** | **1.0** | **114.5** | **N** | **4.0** | **0.3** | **No** | **14** |
| **MoS_2_-rGO/MWCNT** | **1.4** | **N** | **5.8** | **<2.0** | **N** | **No** | **15** |
| **RGO/Ni cotton yarn** | **0.8** | **292.0** | **68.2** | **6.1(stack)** | **1.4(stack)** | **Yes** | **16** |
| **PPY@MnO_2_@RGO yarn** | **0.8** | **68.5** | **N** | **6.0** | **0.8** | **Yes** | **17** |
|  |  |  | **12.4(stack)** | **1.1 (stack)** | **0.2(stack)** |  |  |
| **GCF/MnO_2_** | **1.8** | **N** | **N** | **9.0** | **N** | **No** | **40** |
|  |  |  | **11.1(stack)** | **5.0(stack)** | **0.9(stack)** |  |  |
| **PEDOT/MWNT biscrolled yarns** | **0.8** | **179.0** | **N** | **1.4** | **40.0** | **Yes** | **41** |
| **CuO/AuPd/MnO_2_/Cu wire** | **0.8** | **N** | **N** | **0.6 (stack)** | **0.4(stack)** | **Yes** | **42** |
| **Nickel fiber/Co_3_O_4_** | **1.5** | **N** | **2.1(stack)** | **0.6(stack)** | **1.5(stack)** | **Yes** | **43** |

PPY: polypyrrole; PEDOT: poly(3,4-ethylenedioxythiophene); SWCNT: single-walled carbon nanotube; MWCNT: multiple-walled carbon nanotube. "N" stands for "Not mentioned", "stack" presents that the whole device includes active electrodes, separators and electrolyte.
